# The combination of NDUFS1 with CD4^+^ T cell infiltration predicts favorable prognosis in kidney renal clear cell carcinoma

**DOI:** 10.3389/fcell.2023.1168462

**Published:** 2023-07-04

**Authors:** Dong Wu, Lin He, Zhe Xu, Ruo-Fei Tian, Xin-Yu Fan, Jing Fan, Jie Ai, Hui-Jie Bian, Wei-Jun Qin, Jun Qin, Ling Li

**Affiliations:** ^**1**^ National Translational Science Center for Molecular Medicine, Department of Cell Biology, School of Basic Medicine, The Fourth Military Medical University, Xi’an, China; ^**2**^ Department of Oncology, Tangdu Hospital, The Fourth Military Medical University, Xi’an, China; ^**3**^ Unit 94710 of the PLA, Wuxi, China; ^**4**^ Department of Urology, Xijing Hospital, The Fourth Military Medical University, Xi’an, China

**Keywords:** kidney renal clear cell carcinoma, NDUFS1, survival, hsa-miR-320b, CD4^+^ T cell infiltration

## Abstract

**Background:** Kidney renal clear cell carcinoma (KIRC) is an immunogenic tumor, and immune infiltrates are relevant to patients’ therapeutic response and prognosis. *NDUFS1*, the core subunit of mitochondrial complex I, has been reported to be associated with KIRC patients’ prognosis. However, the upstream regulator for *NDUFS1* and their correlations with immune infiltration remain unclear.

**Methods:** The expression of *NDUFS* genes in KIRC and their influences on patients’ survival were investigated by UALCAN, ENCORI, Oncomine, TIMER as well as Kaplan-Meier Plotter. miRNAs regulating *NDUFS1* were predicted and analyzed by TargetScan and ENCORI. The correlations between *NDUFS1* expression and immune cell infiltration or gene marker sets of immune infiltrates were analyzed *via* TIMER. The overall survival in high/low *NDUFS1* or hsa-miR-320b expressed KIRC patients with or without immune infiltrates were analyzed *via* Kaplan-Meier Plotter. The combined NDUFS1 expression and/or CD4^+^ T cell infiltration on KIRC patients’ overall survival were validated by multiplexed immunofluorescence (mIF) staining in tissue microarray (TMA). Furthermore, the influences of NDUFS1 expression on the chemotaxis of CD4^+^ T cells to KIRC cells were performed by transwell migration assays.

**Results:** We found that the low expression of *NDUFS1* mRNA and protein in KIRC was correlated with unfavorable patients’ survival and poor infiltration of CD4^+^ T cells. In patients with decreased CD4^+^ T cell infiltration whose pathological grade less than III, TMA mIF staining showed that low expression of NDUFS1 had significantly poor OS than that with high expression of NDUFS1 did. Furthermore, hsa-miR-320b, a possible negative regulator of *NDUFS1*, was highly expressed in KIRC. And, low *NDUFS1* or high hsa-miR-320b consistently correlated to unfavorable outcomes in KIRC patients with decreased CD4^+^ T cell infiltration. *In vitro*, *NDUFS1* overexpression significantly increased the chemotaxis of CD4^+^ T cell to KIRC cells.

**Conclusion:** Together, *NDUFS1*, upregulated by decreased hsa-miR-320b expression in KIRC patients, might act as a biomarker for CD4^+^ T cell infiltration. And, the combination of NDUFS1 with CD4^+^ T cell infiltration predicts favorable prognosis in KIRC.

## 1 Introduction

Kidney renal clear cell carcinoma (KIRC), characterized by biallelic loss of function of the von Hippel Lindau (*VHL*) tumor suppressor gene, is the adenocarcinoma derived from the renal tubular epithelial cell. The amount of new KIRC cases is ranking seventh in all cancer types around the world (Siegel et al., 2022). As a deadly or fatal disease, one-third of patients have advanced or metastatic disease at diagnosis; and 30%–40% of patients have tumor recurrence after surgical resection (Toth and Cho, 2020; Singh, 2021). As an important strategy for KIRC, the systemic therapy has successively experienced the 4 stages of development: the precytokine era, the cytokine era, the molecularly targeted era (2005–2014), and the immune checkpoint blockade era (2015 to present), with 2 stages relating to immunotherapy (Hasanov et al., 2020).

In contrast to limited efficacy and serious toxicity of cytokine therapy that adopts high dose of IL-2 and IFN-α, immune checkpoint inhibitors (ICIs) targeting programmed death (PD)-1 or CTLA-4 have improved efficacy and safety greatly. However, a large percentage of KIRC patients fail to obtain complete responses and durable remissions, whether by using ICIs single-agent or the combination of ICIs with tyrosine kinase inhibitors (TKIs) (Hasanov et al., 2020; Wang et al., 2021). The reasons for the resistant mechanisms may include absence of antigenic proteins or defects in antigen presentation, decreased T cell activity, the existence of other inhibitory checkpoints such as VISTA, LAG-3, and TIM-3, and having other immunosuppressive cells like tumor-associated macrophages, or regulatory T cells (Hasanov et al., 2020). Besides these, immune infiltration has been considered as a significant determinant that helps to select optimal patients or to predict therapeutic response and prognosis. For example, it is reported that the recurrence of KIRC was related to lower T cells, while a higher T effector (Teff)/Treg ratio would reduce recurrence rate (Ghatalia et al., 2019). In COMPARZ phase III trial of first-line TKIs sunitinib or pazopanib, patients’ comparably therapeutic effect was sufficiently affected by the immune infiltration level (Laurell et al., 2017). Thus, biomarkers identifying immune cell infiltration are urgently required.

Mitochondrial metabolism is essential for macromolecular synthesis, cell proliferation, cell migration/invasion, cell division or cell differentiation, *etc* (Vasan et al., 2020). By adopting general features of immune-based groupings and incorporating transcriptomic and proteomic features, Clark DJ et al. divided clear cell renal cell carcinoma (ccRCC) cohort into four subtypes: CD8^+^ infiamed, CD8^−^ infiamed, VEGF immune desert and metabolic immune desert (Clark et al., 2019). Metabolic immune desert tumors display low immune scores but elevated mitochondrial, oxidative phosphorylation (OXPHOS) and glycolysis protein expression, indicating that metabolism is tightly associated with immune microenvironment in ccRCC. These findings suggest that cell metabolism is connected to immune signatures, and mitochondrial or OXPHOS protein that associated with immune cell infiltration would be a potential novel biomarker for patient selection or prognosis monitoring.

Mitochondrial complex I (NADH dehydrogenase) is the first and largest mitochondrial enzyme complex of OXPHOS. In many cancers including KIRC (or ccRCC), the dysfunction of complex I resulted in activating Akt pathway and cancer progression (Santidrian et al., 2013; Ellinger et al., 2017). Complex I is composed of 45 subunits in mammals, and 8 of which form a chain of iron-sulfur (Fe/S) clusters that encoded by *NDUFS* [(NADH dehydrogenase (ubiquinone) Fe-S protein)] genes. Their encoded protein, namely, NDUFSs, are responsible to transfer electron from NADH to coenzyme Q or ubiquinone. Although the expression profiles and prognosis values of *NDUFS* genes in KIRC have been reported recently, the relationship between *NDUFS1* and immune cell infiltration in cancer have not been explored. Besides, the detailed role, mechanism and regulation of *NDUFS* genes in cancer progression remain unknown.

In this study, through digging into public online databases, we firstly screened the key player in *NDUFS* genes family based on expression levels and patients’ survival in KIRC; then we explored whether *NDUFS* genes were correlated with immune infiltration and whether their correlation could combinedly predict the patients’ prognosis; Finally we probed the upstream regulator for *NDUFS1* and validated their individual correlations with immune cell infiltration and/or with KIRC patients’ prognosis. Our findings highlight that *NDUFS1*, downregulated by hsa-miR-320b, might act as a biomarker for CD4^+^ T cell infiltration and predict favorable prognosis in KIRC patients. Our understanding of *NDUFS* genes in predicting KIRC immune cell infiltration would help to identify patients that benefited from ICIs and to develop the rational combinations of mitochondrial inhibitors with ICIs for future novel and efficacious anti-cancer treatments.

## 2 Materials and methods

### 2.1 Study design and data collection

As described in the flow chart (Supplementary Figure S1), we screened differentially expressed genes (DEGs) in *NDUFS* gene family and overall survival (OS) analysis of *NDUFS* genes in KIRC by intersecting three online databases, respectively. We further explored NDUFS1 protein expression, its prognostic value and NDUFS1-correlated immune infiltrates in KIRC by digging into UALCAN and conducting TMA multiplexed immunofluorescence (mIF) validation. Meanwhile, possible miRNAs targeting NDUFS1 and NDUFS1-related cuproptosis genes were predicted and analyzed via online databases. Moreover, to analyze the combined prognostic effect of NDUFS1 with miRNAs or cuproptosis-regulated gene in KIRC, we collected mRNA and miRNA sequencing data of TCGA database from Genomic Data Commons (GDC) Data Portal (https://portal.gdc.cancer.gov).

### 2.2 UALCAN database analysis

UALCAN (RRID:SCR_015827) (http://ualcan.path.uab.edu/index.html) is a comprehensive and interactive web resource which is designed to analyze cancer OMICS data including TCGA and CPTAC database, and to evaluate epigenetic regulation of gene expression (Chandrashekar et al., 2017). We used UALCAN for investigating the *NDUFS* genes’ expression profiles in KIRC and the relationship of *NDUFS1* expression with tumor grades, individual cancer stages or KIRC subtypes. Besides, the protein expression and promoter methylation of *NDUFS1* in KIRC were also analyzed.

### 2.3 ENCORI database analysis

ENCORI (RRID:SCR_016303) (http://starbase.sysu.edu.cn/index.php) is an open-source platform for studying miRNA-target interactions, of which 2.5 million miRNA-mRNA interactions are included. ENCORI also provides platforms for analyzing the differential expression and survival of miRNAs and the targeted mRNAs (Li et al., 2014). Herein on this platform, the expression profiles and overall survival of *NDUFS* genes, the predicted miRNAs, and their correlation with patients’ survival time in KIRC were explored.

### 2.4 GEPIA database analysis

GEPIA 2.0 (Gene Expression Profiling Interactive Analysis, RRID:SCR_018294) (http://gepia2.cancer-pku.cn/) is an interactive web server for analyzing the RNA sequencing expression data from the TCGA and the GTEx projects, which provides differential expression analysis, patient survival analysis, etc (Tang et al., 2019). Thus, we acquired the *NDUFS* genes’ expression profiles between normal and cancer tissues and the overall survival data on this platform.

### 2.5 Kaplan-Meier Plotter database analysis

The Kaplan Meier plotter (RRID:SCR_018753) (https://kmplot.com/analysis/) is an online database which is capable to assess the effect of 54,000 genes (mRNA, miRNA, protein) on patients’ survival in 21 cancer types from GEO, EGA, and TCGA (Nagy et al., 2021). We used this database to predict the OS and disease-free survival (DFS) of KIRC patients according to the expression of *NDUFS* genes or hsa-miR-320b and the association of their expression with clinical outcome at different levels of immune infiltrates.

### 2.6 Oncomine database analysis

Oncomine (RRID:SCR_007834) (https://www.oncomine.org/resource/main.html) datasets are composed of microarray data measuring either mRNA expression or DNA copy number in primary tumors, cell lines or xenografts, usually from published research (Rhodes et al., 2007). We use Oncomine database to compare the expression profiles of *NDUFS1* between normal and tumor tissues in different cancer types.

### 2.7 TIMER database analysis

TIMER 2.0 (RRID:SCR_018737) (http://timer.cistrome.org/) is a comprehensive web server for estimating the abundance of immune infiltrates across 32 cancer types from The Cancer Genome Atlas (TCGA) (Li et al., 2020). We applied TIMER for analyzing the following three aspects: the *NDUFS1* expression in different types of cancers and its correlation with the abundance of immune infiltrates including NK, neutrophils, CD4^+^ T, macrophage M1 and NKT cells were explored *via* gene modules; the correlation of *NDUFS1* expression with the gene markers of tumor-infiltrating immune cells was analyzed *via* correlation modules; and the association between immune infiltrates and clinical outcomes was investigated *via* outcome modules.

### 2.8 CProSite database analysis

The cProSite (Cancer Proteogenomic Data Analysis Site) (https://cprosite.ccr.cancer.gov/) is a web-based interactive platform providing visualization of proteomic analysis from the datasets of the National Cancer Institute’s Clinical Proteomic Tumor Analysis Consortium (CPTAC) and National Cancer Institute’s International Cancer Proteogenome Consortium (ICPC). We used cProSite to analyze correlation between the level of NDUFS1 protein and its mRNA or FDX1 protein in KIRC.

### 2.9 TargetScan database analysis

TargetScan (RRID:SCR_010845) (http://www.targetscan.org) can predict biological targets of miRNAs by searching for the presence of conserved 8mer and 7mer sites that match the seed region of each miRNA (Lewis et al., 2005). We used TargetScan to predict the possibly conserved miRNAs that regulate *NDUFS1*.

### 2.10 Tissue microarray (TMA) and multiplexed immunofluorescence (mIF) staining

Human KIRC TMA (Cat.HKidE180Su03, OUTDO BIOTECH, China) were constructed with tumor and paired adjacent tissues from a cohort including 90 primary KIRC patients who were operated between 2006.10 and 2008.2 and were followed for 7–9 years. Patient information including age, gender and follow-up information such as survival times were recorded. For mIF staining, TMA slides were firstly incubated with rabbit monoclonal anti-NDUFS1 antibody (1:200, Cat.ab169540, Abcam, United Kingdom), mouse monoclonal anti-CK antibody (1:400, Cat.PA125, Abcarta, China) and rabbit monoclonal anti-CD4 antibody (1:200, Cat.PA285, Abcarta, China), followed by incubation with goat anti-mouse (1:250, Cat.35502, ThermoFisher Scientific, United States) or goat anti-rabbit (1:500, Cat.A23320, Abbkine, United States) secondary antibodies. The samples were finally counterstained with DAPI and the images were analyzed by TissueFAXS Viewer software. The percentage of NDUFS1 and CK double positive cells in each tissue was described as the expression level of NDUFS1 in KIRC and adjacent normal tissues, and the percentage of CD4 positive cells in each tumor tissues was described as the infiltration level of CD4^+^ T cell in KIRC. For the survival analysis, patients were divided into low or high expression groups which is cut off by mean value of NDUFS1 or CD4 expression in KIRC.

### 2.11 Cell culture and transfection

The human kidney renal clear cell lines ACHN and 786-O, and immortal human CD4 T lymphocytes Jurkat T were purchased from the Shanghai Institutes for Biological Sciences (Shanghai, China). Cells were cultured in DMEM (Cat.11965092, Invitrogen) containing 10% fetal bovine serum (Cat.10100147, Invitrogen), 100 U/mL penicillin, and 100 μg/mL streptomycin (Cat.15240062, Invitrogen). The pcDNA3.1-NDUFS1 plasmid and pcDNA3.1 vector control plasmid were obtained from Tsingke Co., Ltd. (Beijing, China). The transfection was performed by Lipofectamine 2000 (Cat.11668500, Invitrogen).

### 2.12 Quantitative real-time PCR

Total RNA was isolated from KIRC cells using the total RNA kit II (Omega, CA, United States) and was reverse transcribed into complementary DNA by PrimeScriptTM RT reagent kit (TaKaRa Biotechnology, Otsu, Japan). Single stranded complementary DNA was amplified by quantitative real-time PCR (qRT-PCR) using the SYBR Premix ExTaq kit (TaKaRa Biotechnology) in the Mx3005P Real-Time PCR system (Agilent Technologies, Germany). The following primers were used for qRT-PCR analysis: NDUFS1 forward 5′-TTA​GCA​AAT​CAC​CCA​TTG​GAC​TG-3′, reverse: 5′-CCC​CTC​TAA​AAA​TCG​GCT​CCT​A-3′; β-actin forward: 5′-AGC​GAG​CAT​CCC​CCA​AAG​TT-3′, reverse:5′- GGGCACGAAGGCT CATCATT-3′.

### 2.13 Western blot analysis

Western blot analysis was performed according to the standard protocol. Briefly, the proteins were extracted by RIPA (10% PMSF) from KIRC cells, and then were electrophoresed by SDS-PAGE and transferred to PVDF membranes (Millipore). Membranes were blocked in 5% skimmed milk and incubated with anti-NDUFS1 (1:10,000, Cat.Ab169540, Abcam, United Kingdom) and anti-β-actin (1:20,000, Cat.66009-1-Ig, Proteintech, China) at 4°C overnight, and then labeled with anti-mouse IgG (Pierce, IL, United States) or anti-rabbit IgG (Pierce) at room temperature for 1 h, respectively. The protein signal was visualized using a Western-Light chemiluminescent detection system (Image Station 4000 MM Pro, MA, United States of America).

### 2.14 Chemotaxis assays *in vitro*

Chemotaxis assays *in vitro* were performed using transwell chambers (Cat.3413, Corning, United States). A total of 5 × 10^5^ Jurkat T cells in 100 μl DMEM culture medium were placed in the upper chambers, 1 × 10^5^ NDUFS1-overexpressed or control KIRC cells in 300 μl DMEM culture medium were seeded in the lower wells and the chambers were incubated for 24 h at 37°C. The migrated Jurkat T cells on the inserts were stained with crystal violet. Chemotaxis ability was determined by counting the stained cells on the bottom surface of each membrane in 4 random fields, and images were captured at 200×magnification.

### 2.15 Statistical analysis

Pearson or Spearman’s correlation coefficients (R or Rho) and *p*-values were used to evaluate the correlations of *NDUFS1* with miRNAs or immune infiltrates. OS and DFS generated by Kaplan-Meier plots were used to evaluate the prognostic impacts of *NDUFS* genes or miRNAs and their combination with immune infiltrates, and the results are displayed with *p*-values based on a log-rank test and a hazard ratio (HR). Two-tailed Student’s t-tests were used to test the significance of differences between two groups in TMA analysis and chemotaxis assays *in vivo*. *p*-values less than 0.05 were considered statistically significant.

## 3 Results

### 3.1 The expression and survival analysis of NDUFS genes in KIRC

**FIGURE 1:**
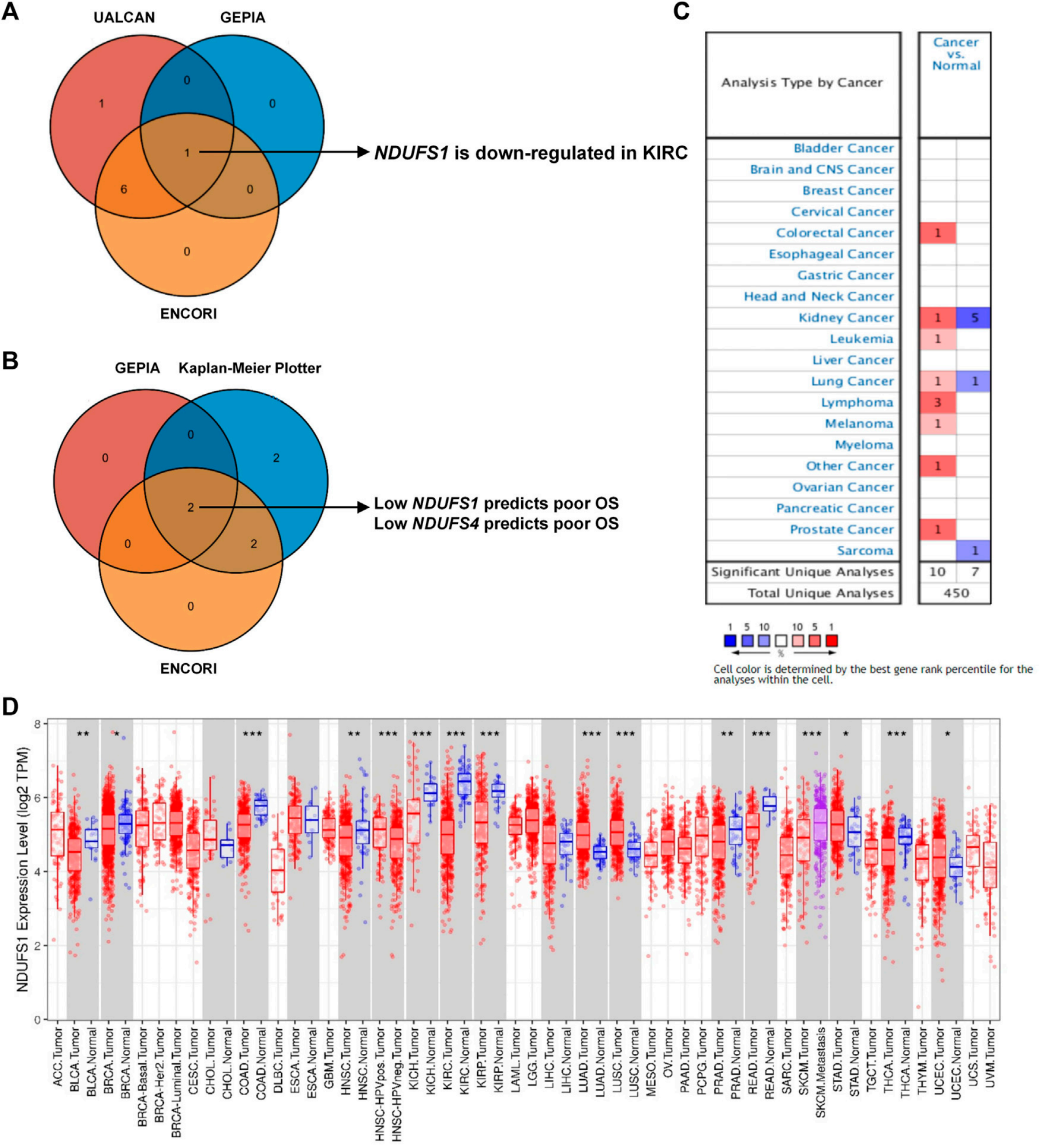
Comprehensive *NDUFS1* mRNA expression profiles in pan-cancer. **(A)** Venn diagram comparison of *NDUFS* genes’ expression among UALCAN, ENCORI and GEPIA database. **(B)** Venn diagram comparison of the association of *NDUFS* genes with patient’s survival among GEPIA, Kaplan-Meier Plotter and ENCORI database. **(C)** *NDUFS1* mRNA expression levels in various cancer tissues comparing with normal tissues were analyzed by the Oncomine database. **(D)** *NDUFS1* expression levels in pan-cancers were analyzed by TIMER database. (*p < 0.05, **p < 0.01, ***p < 0.001).

To figure out the expression of *NDUFS* genes in KIRC patients, we firstly investigated three online databases (Supplementary Figure S2). In UALCAN, *NDUFS5* mRNA level was significantly increased while all other seven *NDUFS* mRNA levels were significantly decreased. In ENCORI, we discovered that *NDUFS5* mRNA level again was significantly increased (*p* = 0.00074), but all other six *NDUFS* mRNA levels except *NDUFS4* were significantly decreased in KIRC tissues as compared to those in normal tissues. When it comes to GEPIA, only *NDUFS1* mRNA level was significantly decreased in KIRC tissues comparing with that in normal tissues. Then, we drew Venn diagrams to summarize the above online databases results. As shown in Figure 1A, *NDUFS1* was the only overlapped *NDUFS* gene that differentially expressed between tumor and normal tissues in KIRC patients among UALCAN, ENCORI and GEPIA databases.

To further explore the association of *NDUFS* genes’ expression with patients’ survival in KIRC, we investigated the OS in patients with high or low expression of *NDUFS* genes (Supplementary Figure S3). From GEPIA, we found that KIRC patients with low expression of *NDUFS1* or *NDUFS4* had significantly poor OS than the patients with high expression did. Meanwhile, Kaplan-Meier Plotter displayed a significantly unfavorable OS in KIRC patients who have lower expression of *NDUFS1* or *NDUFS4*, while a significantly favorable OS in KIRC patients with lower expression of *NDUFS3*, *NDUFS5*, *NDUFS6*, *NDUFS7* or *NDUFS8*. Analysis from ENCORI demonstrated that low expression of *NDUFS1* or *NDUFS4* was associated with patients’ shorter OS; in contrast, the high level of *NDUFS6* or *NDUFS8* was related to longer OS in KIRC patients. Moreover, GEPIA database analysis showed that the KIRC patients with low level of *NDUFS1*, *NDUFS2* or *NDUFS4* had poor DFS than those with high level did (Supplementary Figure S4). Together, the above three databases consistently demonstrated that the expression of *NDUFS1* or *NDUFS4* has a significantly positive correlation with favorable survival in KIRC patients (Figure 1B). Combined with the expression profile of *NDUFS* genes, *NDUFS1* is downregulated in KIRC and low expression of *NDUFS1* is associated with poor prognosis but high expression associated with favorable prognosis.

### 3.2 NDUFS1 expression is negatively correlated with clinicopathological features in KIRC

To extend our analysis of *NDUFS1* mRNA level in KIRC to the universal expression profiles in other cancer types, we further investigated the Oncomine and TIMER databases. Oncomine database revealed that *NDUFS1* mRNA expression was increased significantly in colorectal cancer, leukemia, lymphoma, melanoma and prostate cancer while was decreased significantly in sarcoma (Figure 1C). As for kidney cancer, *NDUFS1* was reported to be increased in 1 study while decreased in 5 studies. In TIMER database, *NDUFS1* was upregulated significantly in LUAD, LUSC, STAD and UCEC but was downregulated significantly in BLCA, BRCA, COAD, HNSC, PRAD, READ, THCA, and KICH, KIRC, KIRP (Figure 1D). These two databases consistently displayed that *NDUFS1* expression was significantly downregulated in the majority of tumor tissues, especially decreased in kidney cancers, including KIRC, KIRP and KICH.

**FIGURE 2:**
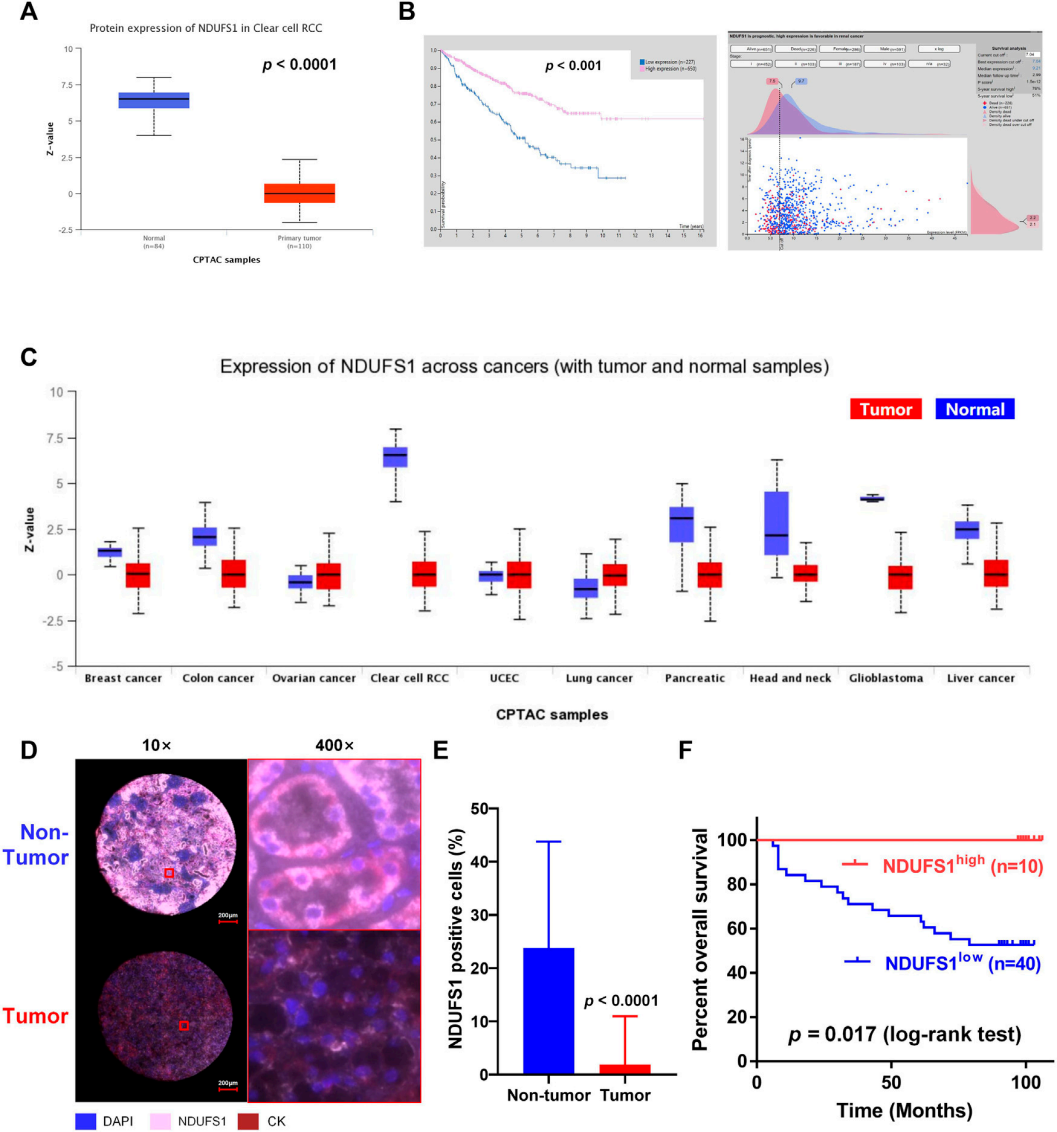
The NDUFS1 protein expression and patient’s overall survival analysis in KIRC. **(A)** The protein expression of NDUFS1 were analyzed by UALCAN database. **(B)** The percent of 5-year survivals in patients with high or low NDUFS1 protein expression were analyzed by Human Protein Atlas database. **(C)** NDUFS1 expression of pan-cancer with tumor and non-tumor samples were analyzed by UALCAN database. **(D)** Representative immunofluorescence images of NDUFS1 positive cells in non-tumor and KIRC tissues. Blue, DAPI; pink, NDUFS1; red, CK. **(E)** The quantitative analysis of NDUFS1 expression in tumor tissues (n = 50) and non-tumor tissues (n = 82) of KIRC TMA. **(F)** The overall survival analysis of KIRC patients with high or low NDUFS1 expression.

As for the protein level, the expression of NDUFS1 in primary KIRC tumor was also significantly decreased as compared to that in normal tissues, and patients with high NDUFS1 expression had significantly favorable survival than those with low expression did (Figures 2A,B). And, 5-year survivals in high or low NDUFS1 expressed patients were 76% or 51% respectively. Besides, we investigated the correlation between protein and mRNA levels of NDUFS1 in KIRC *via* cProSite, and we found that the correlation coefficient in adjacent normal tissue was 0.58 while in tumor was only 0.23 (Supplementary Figure S5A), indicating that *NDUFS1* mRNA in tumor may be further epigenetically modified at post-transcriptional level. In pan-cancer analysis, the protein level of NDUFS1 was significantly decreased in 6 cancers of breast, colon, pancreas, head and neck, glioblastoma and liver, with decreasing most greatly in ccRCC (KIRC) (Figure 2C). Furthermore, in mIF staining of KIRC TMA, we found that the protein level of NDUFS1 was significantly decreased in KIRC tissues as compared to that in normal tissues (*p* < 0.0001, Figures 2D,E). And KIRC patients with low NDUFS1 expression had significantly poor overall survival times than those with high NDUFS1 expression did (*p* = 0.017, Figure 2F). Together, all these data suggested that the expression of both *NDUFS1* mRNA and protein were significantly decreased in KIRC patients and correlated with poor patients’ survival.

Moreover, we analyzed the *NDUFS1* expression based on different tumor grades, patients’ stages and subtypes through digging into UALCAN database. We found that the expression of *NDUFS1* was decreased gradually when the tumor grade was increasing or the individual cancer stage was enhancing, with the lowest NDUFS1 expression occurring in Grade 4 and Stage 4 (Supplementary Figure S5B, C). As far as subtypes were concerned, ccB subtype with poor risk had much lower level of NDUFS1 protein expression than ccA subtype with good risk did (Supplementary Figure S5D). Therefore, lower NDUFS1 expression was correlated with advanced tumor grades, patients’ stages and worse subtype in KIRC.

### 3.3 The low NDUFS1 expression and the associated poor CD4^+^ T cell infiltration combinedly predict unfavorable prognosis in KIRC

**FIGURE 3:**
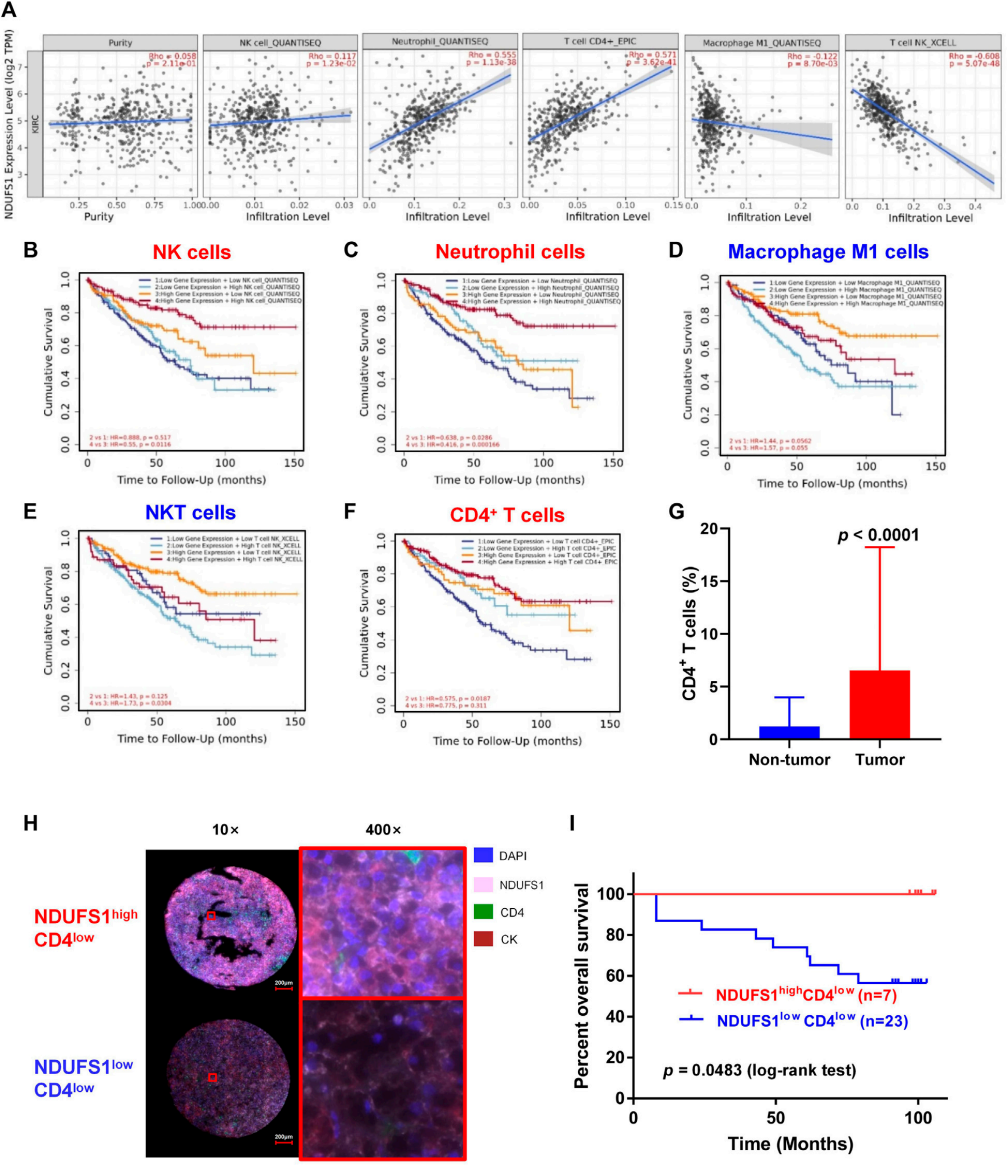
The low NDUFS1 expression and associated poor CD4^+^ T cell infiltration combinedly predict unfavorable survival in KIRC patients. **(A)** Correlation analysis of *NDUFS1* expression with immune infiltration were done by TIMER database. **(B–F)** Survival curves combining high or low *NDUFS1* expression levels with high or low infiltration of immune cells were analyzed by TIMER. **(G)** The quantitative analysis of CD4 expression in tumor tissues (n = 50) and non-tumor tissues (n = 82) by mIF staining in KIRC TMA. **(H)** Representative immunofluorescence images of NDUFS1^high^ CD4^low^ and NDUFS1^low^CD4^low^ cells in KIRC TMA. Blue, DAPI; pink, NDUFS1; green, CD4; red, CK. **(I)** The overall survival analysis in NDUFS1^high^ CD4^low^ and NDUFS1^low^CD4^low^ KIRC patients whose pathological grade less than Ⅲ.

To figure out whether the low *NDUFS1* expression associated poor OS in KIRC patients was related to the altered immune cell infiltration, we then searched the TIMER database. As shown in Figure 3A, *NDUFS1* expression was significantly correlated with the infiltration of NK, neutrophil, CD4^+^ T, macrophage M1 and NKT cells. Judging by the correlation coefficient, however, the *NDUFS1* expression has a moderately positive correlation with the infiltration of neutrophil cells (r = 0.555) and CD4^+^ T cells (r = 0.571), while has a moderately negative correlation with NKT cell infiltration (r = −0.608).

We further explored the expression correlation of *NDUFS1* with marker genes of immune cells (Table 1). Firstly, we found that *NDUFS1* was positively correlated with 4 marker genes in neutrophil cells that including *FCGR3B* (r = 0.302), *SIGLEC5* (r = 0.355), *FPR1* (r = 0.260) and *CD11b* (r = 0.226). Then, a positive correlation between the expression of *NDUFS1* and 5 marker genes in CD4^+^ T cell subtypes was seen, for example, *STAT1* in Th1 cells (r = 0.358), *STAT6* (r = 0.371) and *STAT5A* (r = 0.206) in Th2 cells, as well as *FOXP3* (r = −0.204) and *STAT5B* (r = 0.547) in Treg cells. Also, the expression of *NDUFS1* was positively correlated with *TIM-3* (r = 0.315) while negatively correlated with *GZMB* (r = −0.245) in T cell exhaustion. In addition, *NDUFS1* was positively correlated with *C3AR1* (r = 0.306) in monocyte, *INOS* (r = 0.211) in macrophages, as well as *CD209* (r = 0.231) and *BDCA-4* (r = 0.317) in dendritic cells. And, all above correlations were not affected by tumor purity or patients’ age. Taken together, these results suggested that *NDUFS1* highly expressed KIRC had enriched infiltration of immune cells, for example, neutrophil and CD4^+^ T cells, and that the unfavorable prognosis in *NDUFS1* lowly expressed KIRC might be related to the poor infiltration of these immune cells.

**TABLE 1:** Correlation analysis between *NDUFS1* and immune cell type markers in TIMER database.

Cell type	Gene markers	KIRC						
		None			Purity		Age	
		Correlation coefficient		P	Correlation coefficient	P	Correlation coefficient	P
CD8^+^ T cells	CD8A	−0.055		2.05E-01	−0.032	4.88E-01	−0.053	2.22E-01
	CD8B	−0.100		2.05E-02	−0.088	6.02E-02	−0.098	2.35E-02
T cells (general)	CD3D	−0.171		6.98E-05	−0.154	9.02E-04	−0.171	7.64E-05
	CD3E	−0.142		1.01E-03	−0.126	6.66E-03	−0.142	1.04E-03
	CD2	−0.099		2.20E-02	−0.072	1.21E-01	−0.099	2.27E-02
B cells	FCRL2	−0.126		3.52E-03	−0.106	2.24E-02	−0.128	3.03E-03
	CD19	−0.193		7.31E-06	−0.177	1.35E-04	−0.193	7.71E-06
	MS4A1	−0.054		2.15E-01	−0.011	8.14E-01	−0.055	2.03E-01
	CD19	−0.193		7.31E-06	−0.177	1.35E-04	−0.193	7.71E-06
	CD79A	−0.164		1.46E-04	−0.164	3.97E-04	−0.163	1.62E-04
Monocyte	CD86	0.145		8.18E-04	0.177	1.36E-04	0.145	7.88E-04
	***C3AR1***	***0.306***		***5.19E-13***	***0.318***	***2.76E-12***	***0.306***	***5.50E-13***
	CD115(CSF1R)	0.154		3.63E-04	0.165	3.69E-04	0.155	3.50E-04
TAM	CCL2	0.072		9.63E-02	0.148	1.40E-03	0.072	9.90E-02
	CD68	0.130		2.61E-03	0.108	2.00E-02	0.129	2.89E-03
	IL10	0.121		5.31E-03	0.132	4.62E-03	0.121	5.12E-03
Macrophages	***INOS(NOS2)***	***0.211***		***9.15E-07***	***0.218***	***2.33E-06***	***0.215***	***5.53E-07***
	IRF5	0.136		1.65E-03	0.173	1.93E-04	0.133	2.07E-03
	COX2(PTGS2)	0.061		1.62E-01	0.075	1.09E-01	0.075	1.09E-01
	CD163	0.075		1.09E-01	0.288	2.85E-10	0.297	2.74E-12
	VSIG4	0.133		2.09E-03	0.123	8.15E-03	0.135	1.84E-03
	MS4A4A	0.175		4.96E-05	0.181	9.29E-05	0.174	5.42E-05
Neutrophils	CD66b (CEACAM8)	0.069		1.09E-01	0.056	2.31E-01	0.067	1.24E-01
	***FCGR3B***	***0.302***		***1.01E-12***	***0.297***	***7.97E-11***	***0.303***	***1.04E-12***
	CEACAM3	0.082		5.85E-02	0.070	1.33E-01	0.080	6.61E-02
	***SIGLEC5***	***0.355***		***2.59E-17***	***0.362***	***1.06E-15***	***0.355***	***3.09E-17***
	***FPR1***	***0.260***		***1.05E-09***	***0.270***	***4.09E-09***	***0.260***	***1.11E-09***
	CSF3R	−0.018		6.79E-01	0.004	9.38E-01	−0.018	6.81E-01
	S100A12	0.088		4.32E-02	0.105	2.46E-02	0.086	4.86E-02
	***CD11b(ITGAM)***	***0.226***		***1.30E-07***	***0.242***	***1.49E-07***	***0.226***	***1.46E-07***
	CCR7	−0.057		1.90E-01	−0.053	2.57E-01	−0.059	1.74E-01
NK cells	KIR2DL1	−0.008		8.53E-01	−0.032	4.91E-01	−0.011	7.99E-01
	KIR2DL3	−0.042		3.28E-01	−0.061	1.89E-01	−0.046	2.93E-01
	KIR2DL4	−0.153		3.89E-04	−0.155	8.57E-04	−0.157	2.91E-04
	KIR3DL1	−0.015		7.27E-01	−0.027	5.63E-01	−0.018	6.81E-01
	KIR3DL2	−0.123		4.44E-03	−0.109	1.89E-02	−0.127	3.45E-03
	KIR3DL3	−0.017		6.88E-01	0.012	7.91E-01	−0.019	6.61E-01
	KIR3DL3	−0.017		6.88E-01	0.012	7.91E-01	0.012	7.91E-01
	NCR1	0.188		1.27E-05	0.185	6.55E-05	0.186	1.63E-05
	KIR2DS4	−0.035		4.23E-01	−0.036	4.36E-01	−0.038	3.81E-01
Dendritic cells	***CD209***	***0.231***		***7.01E-08***	***0.234***	***3.61E-07***	***0.232***	***6.81E-08***
	HLA-DPB1	0.092		3.45E-02	0.107	2.15E-02	0.092	3.31E-02
	HLA-DQB1	0.044		3.13E-01	0.072	1.23E-01	0.046	2.88E-01
	HLA-DRA	0.191		8.62E-06	0.214	3.56E-06	0.191	8.85E-06
	BDCA-1(CD1C)	0.163		1.58E-04	0.180	9.76E-05	0.164	1.53E-04
	***BDCA-4(NRP1)***	***0.317***		***6.45E-14***	***0.324***	***1.04E-12***	***0.320***	***4.06E-14***
	CD11C(ITGAX)	−0.004		9.31E-01	0.021	6.52E-01	−0.005	8.99E-01
Th1 cells	T-bet (TBX21)	−0.104		1.58E-02	−0.093	4.67E-02	−0.107	1.36E-02
	STAT4	−0.110		1.09E-02	−0.080	8.62E-02	−0.111	1.08E-02
	***STAT1***	***0.358***		***1.62E-17***	***0.392***	***2.32E-18***	***0.358***	***1.56E-17***
	IFN-γ(IFNG)	−0.100		2.06E-02	−0.082	7.92E-02	−0.100	2.13E-02
	TNF-α(TNF)	−0.008		8.55E-01	0.016	7.32E-01	−0.008	8.52E-01
Th2 cells	GATA3	−0.158		2.45E-04	−0.121	9.53E-03	−0.157	2.70E-04
	***STAT6***	***0.371***		***7.48E-19***	***0.351***	***8.38E-15***	***0.370***	***1.18E-18***
	***STAT5A***	***0.206***		***1.62E-06***	***0.255***	***2.65E-08***	***0.207***	***1.49E-06***
	IL13	−0.217		4.04E-07	−0.178	1.23E-04	−0.221	2.79E-07
Tfh cells	BCL6	0.012		7.76E-01	0.025	5.99E-01	0.012	7.89E-01
	IL21	0.012		7.83E-01	0.016	7.27E-01	0.008	8.57E-01
Th17 cells	STAT3	0.008		8.57E-01	0.537	8.45E-36	0.514	4.55E-37
	IL17A	−0.019		6.66E-01	0.031	5.01E-01	−0.025	5.73E-01
Treg	***FOXP3***	***−0.204***		***2.03E-06***	***−0.180***	***1.04E-04***	***−0.205***	***1.93E-06***
	CCR8	0.074		8.69E-02	0.109	1.91E-02	0.075	8.53E-02
	***STAT5B***	***0.547***		***5.54E-43***	***0.547***	***2.91E-37***	***0.549***	***4.66E-43***
	TGF-β(TGFB1)	−0.031		4.72E-01	−0.040	3.90E-01	−0.032	4.68E-01
T cell exhaustion	PD-1(PDCD1)	−0.154		3.76E-04	−0.126	6.76E-03	−0.151	4.64E-04
	CTLA4	−0.124		4.13E-03	−0.077	9.96E-02	−0.124	4.26E-03
	LAG3	−0.194		6.22E-06	−0.172	2.14E-04	−0.193	7.70E-06
	***TIM-3(HAVCR2)***	***0.315***		***9.00E-14***	***0.323***	***1.11E-12***	***0.314***	***1.32E-13***
	***GZMB***	***−0.245***		***1.06E-08***	***−0.251***	***4.98E-08***	***−0.247***	***8.00E-09***

Gene markers with absolute values of correlation coefficient greater than 0.2 and p-values less than 0.05 were bolded.

We further investigated the influence of combined *NDUFS1* expression and immune cell infiltration on KIRC patients’ prognosis by analyzing the OS. We found that in *NDUFS1* highly expressed KIRC patients, poor NK cells (HR = 0.55, *p* = 0.0116, Figure 3B) or neutrophil cells (HR = 0.416, *p* = 0.000166, Figure 3C) was connected to unfavorable survival; while poor macrophage M1 cells (HR = 1.57, *p* = 0.055, Figure 3D) or NKT cells (HR = 0.173, *p* = 0.0304, Figure 3E) was associated with favorable survival. When it comes to the low *NDUFS1* expression groups, KIRC patients with poor neutrophil cells (HR = 0.638, *p* = 0.0286) or CD4^+^ T cells (HR = 0.575, *p* = 0.0187, Figure 3F) had a shorter survival time; while KIRC patients with poor Macrophage M1 cells (HR = 1.44, *p* = 0.0562) had a longer survival time. Combined together, for CD4^+^ T cells that have moderately positive correlation with *NDUFS1* expression, lower immune infiltration may further exacerbate poor survival in lower *NDUFS1* expressed patients. For neutrophils cells, the unfavorable prognosis of neutrophils infiltration in KIRC was independent of *NDUFS1* expression; however, low *NDUFS1* expression and low neutrophils infiltration would have superimposed effects on patients’ survival. Therefore, the combination of *NDUFS1* with CD4^+^ T cell infiltration would improve the efficacy in predicting KIRC patients’ prognosis.

We then validated the above results by conducting mIF staining in KIRC TMA, we found that CD4 expression was significantly increased in KIRC tissues as compared to that in normal tissues (*p* < 0.0001, Figure 3G). More importantly, in patients with decreased CD4^+^ T cell infiltration whose pathological grade less than III, low expression of NDUFS1 had significantly poor OS than that with high expression of NDUFS1 did (*p* = 0.0483, Figures 3H,I), which was consistent to the results of bioinformatic analysis. To reveal the association of NDUFS1 expression with clinicopathological features in KIRC patients, we performed univariate and multivariate Cox proportional hazard analysis of independent predictors for KIRC patients’ overall survival based on clinicopathological features of TMA (Tables 2, 3). We found that advanced age, high AJCC stage, low expression of NDUFS1 and the combination of low NDUFS1 with low CD4 were risk factors for KIRC patients in univariate Cox proportional hazard analysis. After eliminating the influence of other factors by multivariate Cox proportional hazard analysis, we found that only advanced age, low NDUFS1, and the combination of low NDUFS1 with low CD4 were independent risk factors for KIRC patients’ worse prognosis.

**TABLE 2:** The univariate Cox proportional hazard analysis of independent predicators for overall survival of KIRC patients.

Clinicopathological features	HR (95% CI)^a^	*p*-Value
Age (≤65, >65 years old)	0.375 (0.149–0.947)	***0.038***
Gender (Male, Female)	1.071 (0.402–2.855)	0.891
Tumor grade (I + II, III + IV)	0.358 (0.127–1.008)	0.052
AJCC stage (1, 2 + 3+4)	0.362 (0.139–0.942)	***0.037***
NDUFS1 (low, high)	9.554 (1.268–71.955)	***0.028***
NDUSF1^low^CD4^low^, NDUSF1^high^CD4^low^	8.139 (1.055–62.783)	***0.044***

^a^ HR, hazard ratio; CI, confidence interval.

*p*-values less than 0.05 were bolded.

**TABLE 3:** The multivariate Cox proportional hazard analysis of independent predicators for overall survival of KIRC patients.

Clinicopathological features	HR (95% CI)^a^	*p*-Value
Age (≤65, >65 years old)	0.186 (0.064–0.542)	***0.002***
NDUFS1 (low, high)	16.70 (2.095–133.169)	***0.008***
NDUSF1^low^CD4^low^ NDUSF1^high^CD4^low^	43.910 (3.524–547.046)	***0.003***

^a^ HR, hazard ratio; CI, confidence interval.

*p*-values less than 0.05 were bolded.

**FIGURE 4:**
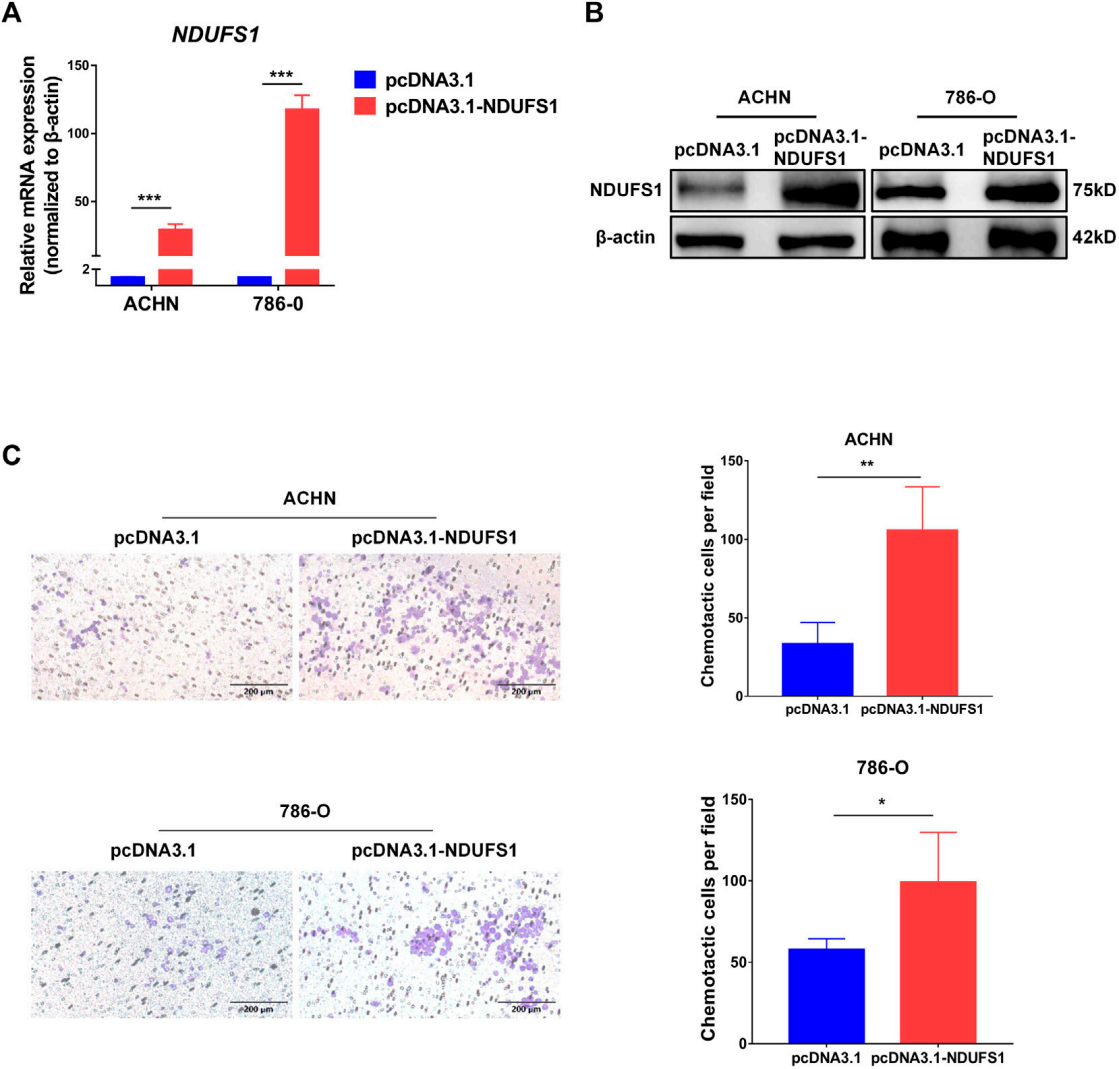
*NDUFS1* overexpression promotes T cell chemotaxis to KIRC cells. **(A, B)** qRT-PCR and Western blot analysis of NDUFS1 mRNA **(A)** and protein **(B)** expression in KIRC cells that transfected with NDUFS1 cDNA. **(C)** The effect of NDUFS1 overexpression on CD4^+^ Jurkat T cell chemotaxis to KIRC cells (*p < 0.05, **p < 0.01, ***p < 0.001).

Moreover, we performed *in vitro* transwell migration assays to analyze the influences of NDUFS1 expression on T cell chemotaxis to KIRC cells. We found that the overexpression of NDUFS1 significantly promoted the migration of CD4^+^ Jurkat T cells to ACHN and 786-O KIRC cells (Figures 4A–C).

### 3.4 Low expression of both NDUFS1 and FDX1 is associated with worse outcome in KIRC patients

**FIGURE 5:**
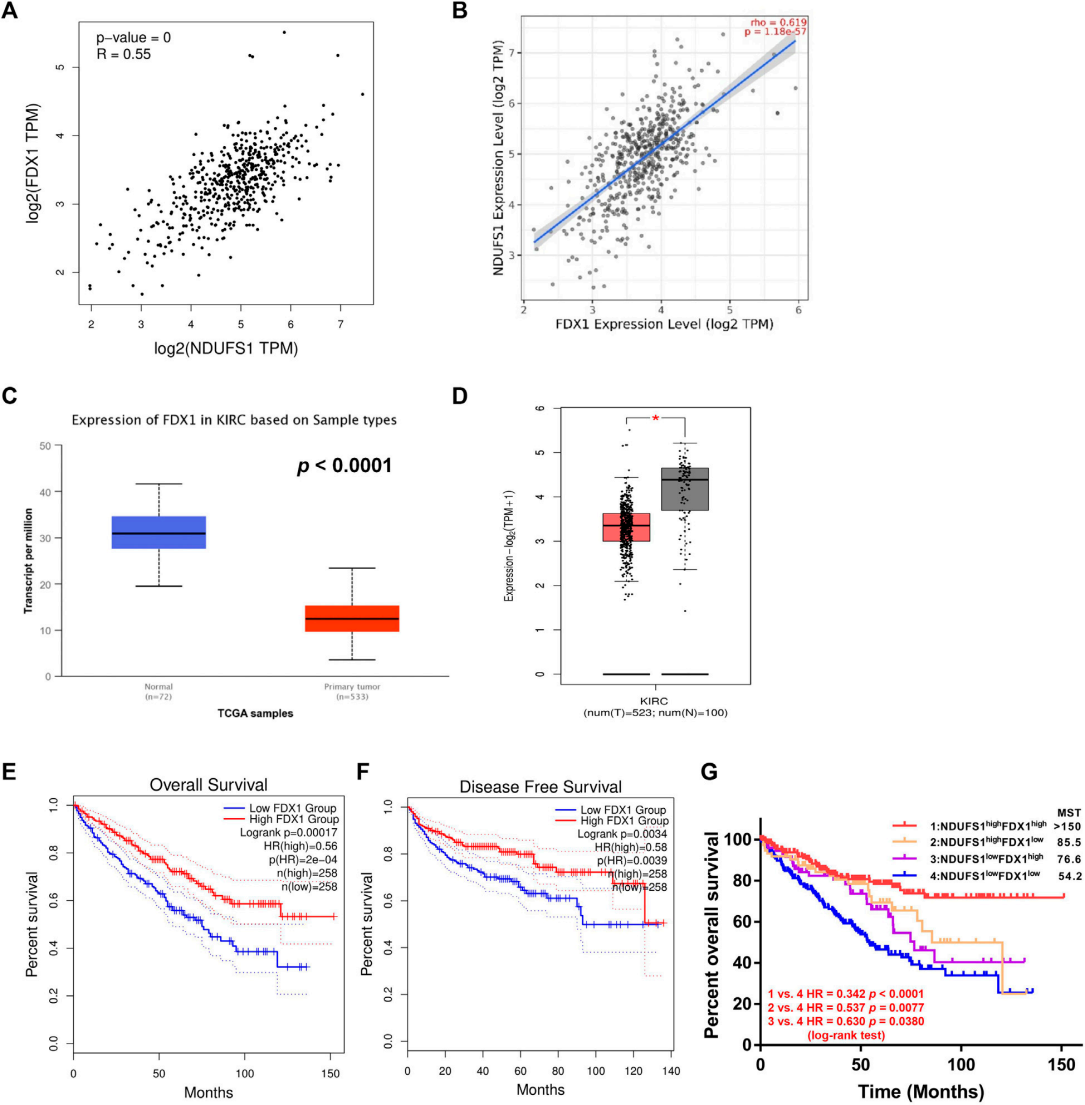
Low expression of both *NDUFS1* and *FDX1* are associated with worse outcomes in KIRC patients. **(A, B)** The correlation between *NDUFS1* and *FDX1* mRNA expression was analyzed by GEPIA **(A)** and TIMER **(B)** database respectively. **(C, D)** *FDX1* mRNA expression was analyzed in UALCAN **(C)** and GEPIA **(D)** database. **(E, F)** The overall survival **(E)** and disease free survival **(F)** analysis in KIRC patients with high or low FDX1 expression was conducted in GEPIA database. **(G)** The overall survival analysis in KIRC patients with combined expression of *NDUFS1* and FDX1. MST, median survival time.

In order to further elucidate potential cellular mechanism of NDUFS1 in affecting KIRC progression, we focused on the key regulator of cuproptosis, namely, FDX1, which is reported to be regulated by mitochondrial respiration. From GEPIA and TIMER database, we found that FDX1 was significantly correlated with NDUFS1 on the mRNA levels in KIRC respectively (R = 0.55, *p* = 0, Figure 5A; R = 0.619, *p* = 1.18e-57; Figure 5B). As for protein level, NDUFS1 and FDX1 was also positively correlative with the correlation coefficient in tumor was only 0.40 while in adjacent normal tissues was 0.82 (Supplementary Figure S5E), which was consisting with the trend between the difference in NDUFS1 mRNA and protein.

Moreover, we found that FDX1 in KIRC tissues was considerably downregulated as compared to that in normal tissues through digging into UALCAN and GEPIA database respectively (P < 1E-12, Figure 5C; *p* < 0.05; Figure 5D). Further analysis from GEPIA database displayed that KIRC patients with low FDX1 had significantly poor OS (*p* = 0.00017, Figure 5E) and DFS (*p* = 0.0034, Figure 5F) than patients with high expression did. By analyzing the downloaded gene expression profiles and corresponding clinical information from TCGA database, we found that patients in NDUFS1^low^FDX1^low^ group had significantly worse prognosis than those in NDUFS1^low^FDX^high^ group (HR = 0.630, *p* = 0.0380, Figure 5G) or those in NDUFS1^high^FDX^low^ group (HR = 0.537, *p* = 0.0077, Figure 5G). In comparison with using either NDUFS1 or FDX1 as a single marker, the combination of low FDX1 and low NDUFS1 predict significantly worse outcome. Together, bioinformatic analysis in a series of online databases have revealed that FDX1, highly related to NDUFS1, was downregulated in KIRC; and low expression of FDX1 was associated with poor prognosis in a similar trend consistent with NDUFS1.

### 3.5 NDUFS1 expression is probably downregulated by hsa-miR-320b in KIRC

**FIGURE 6:**
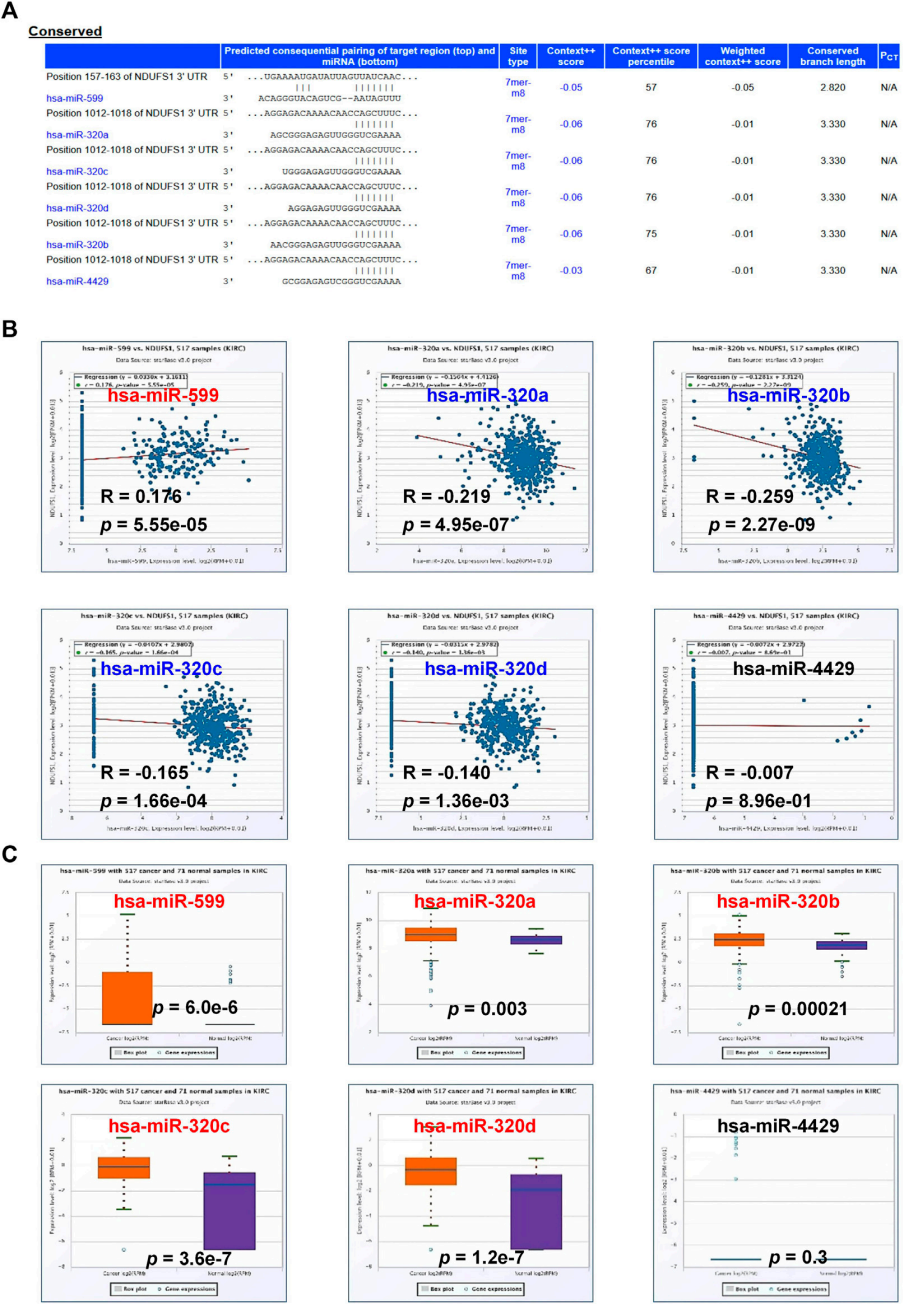
The expression of miRNAs and their correlation with *NDUFS1* in KIRC. **(A)** miRNAs involving in regulating *NDUFS1* were predicted by TargetScan database. **(B)** The correlations between the expression of miRNAs and *NDUFS1* were analyzed by ENCORI. **(C)** The expression levels of miRNAs in KIRC were analyzed by ENCORI.

Considering the low correlation between NDUFS1 protein and mRNA levels in tumor, as well as the fact that the decreased promoter methylation of NDUFS1 was not consistent to the low expression of NDUFS1 mRNA in KIRC (Supplementary Figure S6), we speculated that the expression level of NDUFS1 was probably regulated at the post-transcriptional level. As recent studies showed that mitochondrial biogenesis was in part modified by miRNA silence, resulting in decreased mtDNA expression (Zhang and Xu, 2016). Thus, we tried to explore the potential miRNAs for down-regulating the expression of NDUFS1 in KIRC by digging into TargetScan database. There were 6 conserved miRNAs, including hsa-miR-599, hsa-miR-320a, hsa-miR-320b, hsa-miR-320c, hsa-miR-320d and hsa-miR-4429, were predicted to bind to the 3’ UTR region of NDUFS1 mRNA (Figure 6A). Among them, the level of hsa-miR-599 was positively correlated (r = 0.176, *p* = 5.55e-05) while hsa-miR-320b (r = −0.259, *p* = 2.27e-09), hsa-miR-320a (r = −0.219, *p* = 4.95e-07), hsa-miR-320c (r = −0.165, *p* = 1.66e-04) and hsa-miR-320d (r = −0.140, *p* = 1.36e-03) were negatively correlated with the level of NDUFS1 mRNA in KIRC (Figure 6B). The differential expression analysis demonstrated that hsa-miR-599 (*p* = 6.0e-6), hsa-miR-320a (*p* = 0.003), hsa-miR-320b (*p* = 0.00021), hsa-miR-320c (*p* = 3.6e-7) and hsa-miR-320d (*p* = 1.2e-7) were significantly upregulated in KIRC tissues as compared with those in normal tissues (Figure 6C).

**FIGURE 7:**
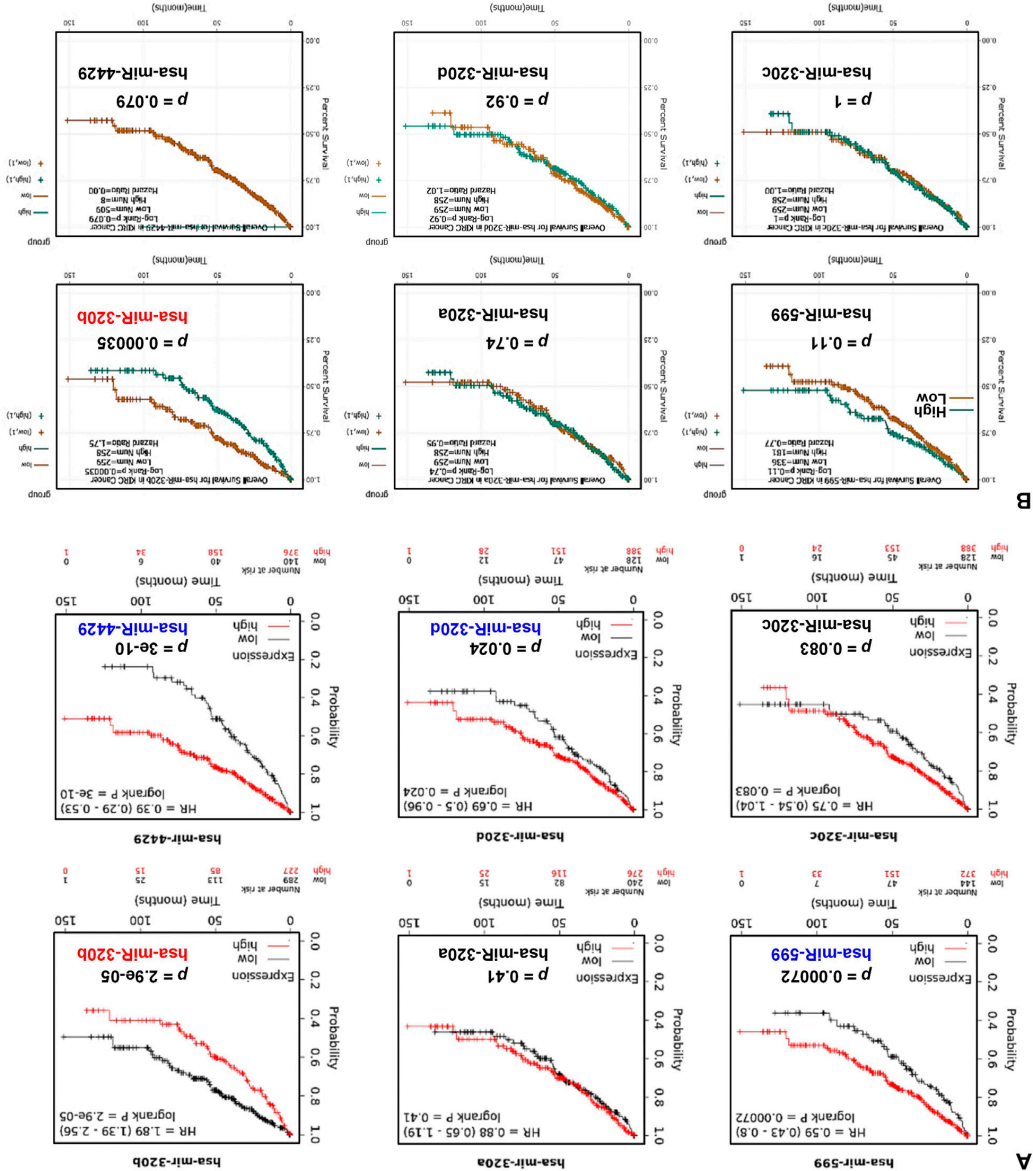
The overall survival analysis of miRNAs in KIRC patients. **(A, B)** The overall survival analysis in KIRC patients with high or low expression of predicted miRNAs were conducted by Kaplan-Meier Plotter **(A)** and ENCORI **(B)**.

**FIGURE 8:**
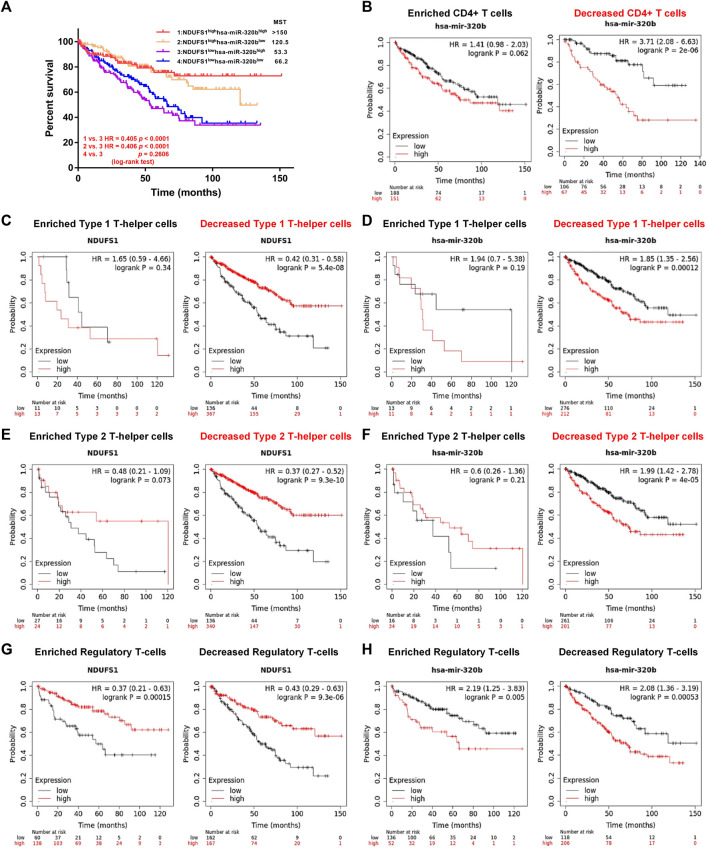
Low *NDUFS1* or high hsa-miR-320b is correlated to poor clinical outcomes in KIRC patients with decreased CD4^+^ T cells infiltration. **(A)** The overall survival analysis in KIRC patients with combined expression of hsa-miR-320b and *NDUFS1*. **(B)** The overall survival analysis in high or low has-miR-320b expressed KIRC patients were analyzed by Kaplan-Meier Plotter based on CD4^+^T cell infiltration. **(C, D)** The overall survival curves in high/low *NDUFS1* **(C)** or high/low has-miR-320b **(D)** expressed KIRC patients based on Th1 cell infiltration. **(E, F)** The overall survival cures in high/low *NDUFS1* **(E)** or high/low has-miR-320b **(F)** expressed KIRC patients based on Th2 cell infiltration. **(G, H)** The overall survival curves in high/low *NDUFS1* **(G)** or high/low has-miR-320b **(H)** expressed KIRC patients based on regulatory T-cell infiltration.

Next, the association of these 6 miRNAs with KIRC patients’ OS were analyzed. Kaplan-Meier Plotter showed that the patients with high level of has-miR-320b had unfavorable OS (*p* = 2.9e-05, Figure 7A) in contrast to the favorable prognosis in KIRC patients with high expression of hsa-miR-599 (*p* = 0.00072), hsa-miR-320d (*p* = 0.024), and hsa-miR-4429 (P = 3e-10). In ENCORI, only high expression of has-miR-320b (*p* = 0.00035) was associated with shorter OS in KIRC whereas other miRNAs had no statistical correlation with KIRC patients’ OS (Figure 7B). Moreover, by analyzing mRNA and miRNA expression profiles and the corresponding clinical information in TCGA database, we found that patients in NDUFS^low^hsa-miR-320b^high^ group had significantly shorter median survival times than those in NDUFS1^high^hsa-miR-320b^high^ group did (53.3 months vs. >150 months, HR = 0.405, *p* < 0.0001, Figure 8A) but not those in NDUFS1^low^hsa-miR-320b^low^ group did. In comparison with using hsa-miR-320b as a single marker, the combination of low NDUFS1 and high hsa-miR-320b predicted significantly worse outcome. To summarize, although 6 miRNAs were predicted to probably affect the transcription of NDUFS1, only hsa-miR-320b was the one that was expressed at high levels, correlated to poor OS and had consistent prognostic values with those of low NDUFS1 expression in KIRC patients. Therefore, hsa-miR-320b was possibly served as an upstream regulator for NDUFS1 in KIRC.

### 3.6 Low NDUFS1 or high hsa-miR-320b is correlated to poor outcomes in KIRC patients with decreased CD4^+^ T cell infiltration

We further explored the correlation of hsa-miR-320b expression with patients’ survival based on immune cell infiltration in KIRC. We found that in KIRC patients with decreased CD4^+^ T cells, high hsa-miR-320b was correlated with poor OS (*P* = 2e-06, Figure 8B), whose prognostic roles in KIRC was consistent to low *NDUFS1*. When it comes to the subgroup of CD4^+^ T cells, low *NDUFS1* (*p* = 5.4e-08, Figure 8C; *p* = 9.3e-10; Figure 8E) or high hsa-miR-320b (*p* = 0.00012, Figure 8D; *P* = 4e-05; Figure 8F) both were associated with poor OS in KIRC patients with decreased Th1 and Th2 cells, respectively. However, in KIRC patients whether with enriched or with decreased Treg cells, low *NDUFS1* or high hsa-miR-320b was all associated with poor OS (Figures 8G,H), suggesting that the prognostic value of *NDUFS1* or hsa-miR-320b was not connected to Treg cell infiltration, but might be related to Th1 and Th2 cell infiltration. To sum up, the expression of low *NDUFS1* or high hsa-miR-320b was connected to poor prognosis in KIRC patients with decreased CD4^+^ T cell infiltration, especially in those with decreased Th1 and Th2 cell infiltration.

## 4 Discussion

In this study, we have explored the potential biomarker associated immune cell infiltration in a view of mitochondrial metabolism, and our findings indicated that *NDUFS1*, a core subunit of mitochondrial complex I whose expression was probably downregulated by hsa-miR-320b and correlated with cuproptosis, might act as a biomarker for CD4^+^ T cell infiltration in KIRC. And, NDUFS1 expression promoted CD4^+^ T cell *in vitro* chemotaxis to KIRC cells. Moreover, the combined low NDUFS1 expression with poor CD4^+^ T cell infiltration predicts unfavorable prognosis. Together, we speculated that NDUFS1 may reprogram the tumor immune microenvironment *via* affecting cell metabolism.

The above findings were obtained by bioinformatics digging and biological experiment validation. For *NDUFS1* mRNA or protein expression and its influence on patients’ survival, both online database and TMA mIF analysis consistently supported that NDUFS1 was downregulated in KIRC and low NDUFS1 was associated with unfavorable survival. As regards to relationship between the combined NDUFS1 expression with CD4^+^ T cell infiltration and patients’ survival, bioinformatics analysis revealed that low *NDUFS1* mRNA expression correlated poor CD4^+^ T cell infiltration was related to unfavorable survival in all KIRC patients; while TMA quantitative data revealed that low NDUFS1 protein expression correlated decreased CD4^+^ T cell infiltration predicted unfavorable prognosis only in KIRC patients whose pathological stage less than III. Based on TMA mIF analysis in the protein level, we hypothesized that NDUFS1 might serve as a prognostic biomarker that predicts better outcome for CD4^+^ T cell infiltration in KIRC patients whose pathological stage less than III. However, due to limited sample size, further larger sample size of KIRC cohorts is required.

A few studies have demonstrated the expression and corresponding function of *NDUFS* genes in the tumor. As prognostic factors, patients with high expression of *NDUFS8* in NSCLC or acute myeloid leukemia has poor overall survival (Su et al., 2016; Wei et al., 2020). However, the opposite prognostic trends are reported for *NDUFS3* and *NDUFS5*. In serous ovarian adenocarcinoma, the downregulation of *NDUFS3* is related to advanced tumor stage and shorter overall survival (Wang et al., 2013); but in invasive breast carcinoma whose upregulation is positively aggressiveness-correlated biomarker signature (Suhane et al., 2011). Besides, the elevated level of *NDUFS5* is associated with good survival in lung adenocarcinoma, but related to significantly reduced time to first progression in gastric cancer (Sotgia and Lisanti, 2017). In the view of drug targets, *NDUFS4* or *NDUFS7* is reported as the targeted gene by casticin or nebivolol for its role in inhibiting cell migration and invasion in mouse melanoma B16F10 cells (Shih et al., 2017), or in blocking complex I activity related colon and breast tumor growth (Nuevo-Tapioles et al., 2020), respectively. As an interacting partner with mitochondrial OSMR or CD147, *NDUFS1/2* or *NDUFS6* confers their individually roles in mediating oxidative phosphorylation related IR resistance of brain tumor stem cells (Sharanek et al., 2020), or in regulating complex I activity and apoptosis in human melanoma (Luo et al., 2014), respectively. As far as the molecular function is concerned, *NDUFS2* silencing inhibits mitochondrial complex I activity and dramatically decreases tumor growth and metastasis rates in lung cancer (Liu L. et al., 2019), and *NDUFS4* knockdown in melanoma cells results in decreased oxidative metabolism, significant decreased CD8^+^ T cell numbers but with superior functional, and an increased response to anti-PD-1 therapy (Najjar et al., 2019). However, the detailed function or mechanism of action for *NDUFS1*, and its relationship with KIRC immunotherapy or immune infiltrates, remain unclear. Although there were similar articles indicating the possible prognostic role of *NDUFS1* in KIRC (Ellinger et al., 2017; Stein et al., 2019), our research for the first time suggested the connection of *NDUFS1* with immune cell infiltrates. Moreover, their combination could predict the patient’s clinical outcomes. Due to the fact that high *NDUFS1* expression predicts favorable prognosis in KIRC, we speculate that the *NDUFS1* associated favorable prognosis may be correlated with immune cell infiltration.

Currently, the possible interventions for *NDUFS1* include gene expression intervention such as overexpression, RNA interference or CRISPR/Cas9-mediated knockout, and small molecule inhibitors. For exploring *NDUFS1*’s function using gain-of-function strategy, the overexpression of *NDUFS1* were used to investigate its role in mediating the radiosensitization of colorectal cancer (Shi et al., 2021) or in miR-3130-5p mediated invasiveness of lung adenocarcinoma (Zhan et al., 2021). In addition, loss-of-function strategy using siRNA or shRNA mediated RNA interference were used to reveal *NDUFS1*-mediated colorectal cancer cell proliferation and tumorigenesis (Ren et al., 2023), or to study the MDM2-binding associated ROS production (Elkholi et al., 2019), respectively. Besides, sgRNA mediated knockout strategy were utilized to validate its necessity in maintaining cancer cell survival under low pH (Michl et al., 2022). For *NDUFS1* as diagnostic or therapeutic target, a near-infrared (NIR) small-molecule fluorophore dye IR-34, directly cleaves NDUFS1 and disrupts electron transporting in the respiratory chain, could be a potentially useful multifunctional theranostic agent for cancer cell targeting, NIR imaging, and therapeutic ER stress inducing (Wang et al., 2018). More interestingly, by exploring the global gene expression and docking profiling of COVID-19 infection, Jabeen, A et al. found that herbal drugs (apigenin, quercetin, and resveratrol) has the potentials to bind and inhibit NDUFS1 (Jabeen et al., 2022), which suggesting these drugs could be possible inhibitors for NDUFS1.

At present, potential companion diagnostic biomarkers involving gene expression are approved in clinics to identify optimal patients or to screen individual treatment. Besides the biomarkers on tumor cells, the future of prognostic biomarker is likely to rely on the components of the tumor microenvironment. As the main cellular components in tumor microenvironment, the cellular composition and functional state of tumor-infiltrating immune cells vary considerably across tumors. In KIRC that contains a large number of immune infiltrates, consisting of NK, DC, T cells, macrophages, etc., the type or the status of immune infiltrates is varied and needed to be predicted. Consistent with facts that *NDUFS4* mediated oxidative metabolism acts as a barrier to the response of PD-1 blockade in melanoma (Najjar et al., 2019), and more and more studies convey the concept that tumor cell metabolites epigenetically regulate the immune cell phenotype (Jiang et al., 2020). Therefore, we thus focused on the prediction of immune infiltrates by mitochondrial metabolism. And, our analysis in this paper revealed that *NDUFS1* could be a metabolic indicator for CD4^+^ T and neutrophil cell infiltrations in KIRC, and the low *NDUFS1* correlated decreased CD4^+^ T predicted unfavorable prognosis. At present, researches on the detailed influences of *NDUFS1* on the infiltration of CD4^+^ T especially Th1 and Th2 cells in KIRC were ongoing.

In KIRC, the significance of miRNA modifications and their prognostic prediction have been reported in bioinformatic analysis (Christinat and Krek, 2015; Liu S. et al., 2019). Moreover, some miRNAs engaging in the occurrence and development of KIRC are identified (Wang et al., 2019; Xue et al., 2019). In this paper, we found that hsa-miR-320b was negatively correlated with *NDUFS1* and predicted poor prognosis of KIRC patients when expressed at high level. And, we further revealed that low *NDUFS1* or high hsa-miR-320b consistently correlated to unfavorable outcomes in KIRC patients with decreased CD4^+^ T cell infiltration. Although our analysis in this paper suggested that hsa-miR-320b could be a potential upstream down-regulator for *NDUFS1*, further validated experiment is still needed. Interestingly, hsa-miR-320b has been recently reported to be a potential biomarker for predicting the efficacy of immunotherapy in advanced NSCLCs (Peng et al., 2020), which partly supporting our finding that hsa-miR-320b is associated with immune infiltrates. Therefore, hsa-miR-320b alone or in combination with its potential target *NDUFS1* could be a miRNA-mitochondrial signature for KIRC immunotherapy.

Recently, a study released on Science has revealed that mitochondrial respiration regulates cuproptosis in the process of which *FDX1* served as the key regulator (Tsvetkov et al., 2022). As for targeting tumor cells, Jiang et al. (Jiang et al., 2022) has reviewed three typical copper induced tumor cell death mechanisms, including oxidative stress, proteasome inhibition and antiangiogenesis, from which oxidative stress produced by mitochondria was considered the most critical and effective tumor-killing method. Additionally, Yao, et al. (Yao et al., 2023) revealed that *FDX1* may serve as an independent factor affecting the prognosis of KIRC, and was correlated with CD4^+^ T cell infiltration. Hence, we tried to explore possible relationship between *NDUFS1* and cuproptosis in KIRC. We found that *FDX1*, decreased in KIRC, was positively related with *NDUFS1* and predicted poor prognosis when expressed at low level. Therefore, our findings indicated *NDUFS1* may correlate to KIRC cuproptosis, which may be served as a possible mechanism of *NDUFS1* in regulating KIRC cells.

From the view of immune-epigenetic-metabolism, we have obtained a preliminary understanding of the possible functions and upstream regulator of *NDUFS1* in KIRC immune infiltration. Nevertheless, our data are mostly derived from dynamically updated online databases, thus more or less leading to possibly unstable results. And, the sample size in TMA with qualified mIF images and available survival information was small. Moreover, the regulation of hsa-miR-320b on *NDUFS1* or *NDUFS1* on cuproptosis are still needed to be further investigated *via in vitro* and *in vivo* biological experiments.

## Data Availability

The datasets presented in this study can be found in online repositories. The names of the repository/repositories and accession number(s) can be found in the article/Supplementary Material.
