# Supplementary figures and images for: The combination of NDUFS1 with CD4+ T cell infiltration predicts favorable prognosis in kidney renal clear cell carcinoma
Source: Front Cell Dev Biol. 2023 Jul 4;11:1168462. doi: 10.3389/fcell.2023.1168462 (PMC10352660; doi:10.3389/fcell.2023.1168462)

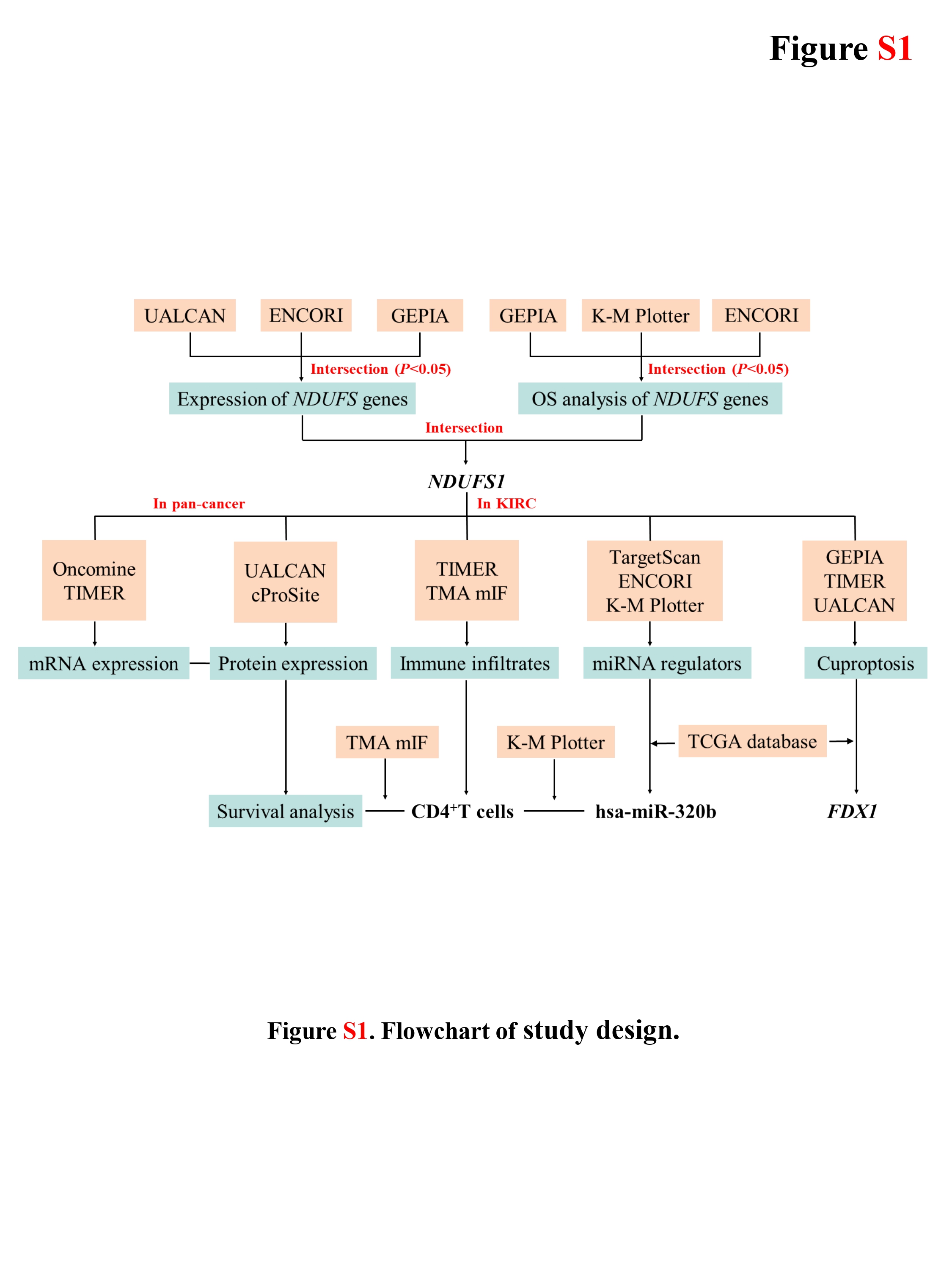

Supplement: Supplementary file 1 [file Presentation1.ZIP › supplementary figures/Figure S1.jpg]

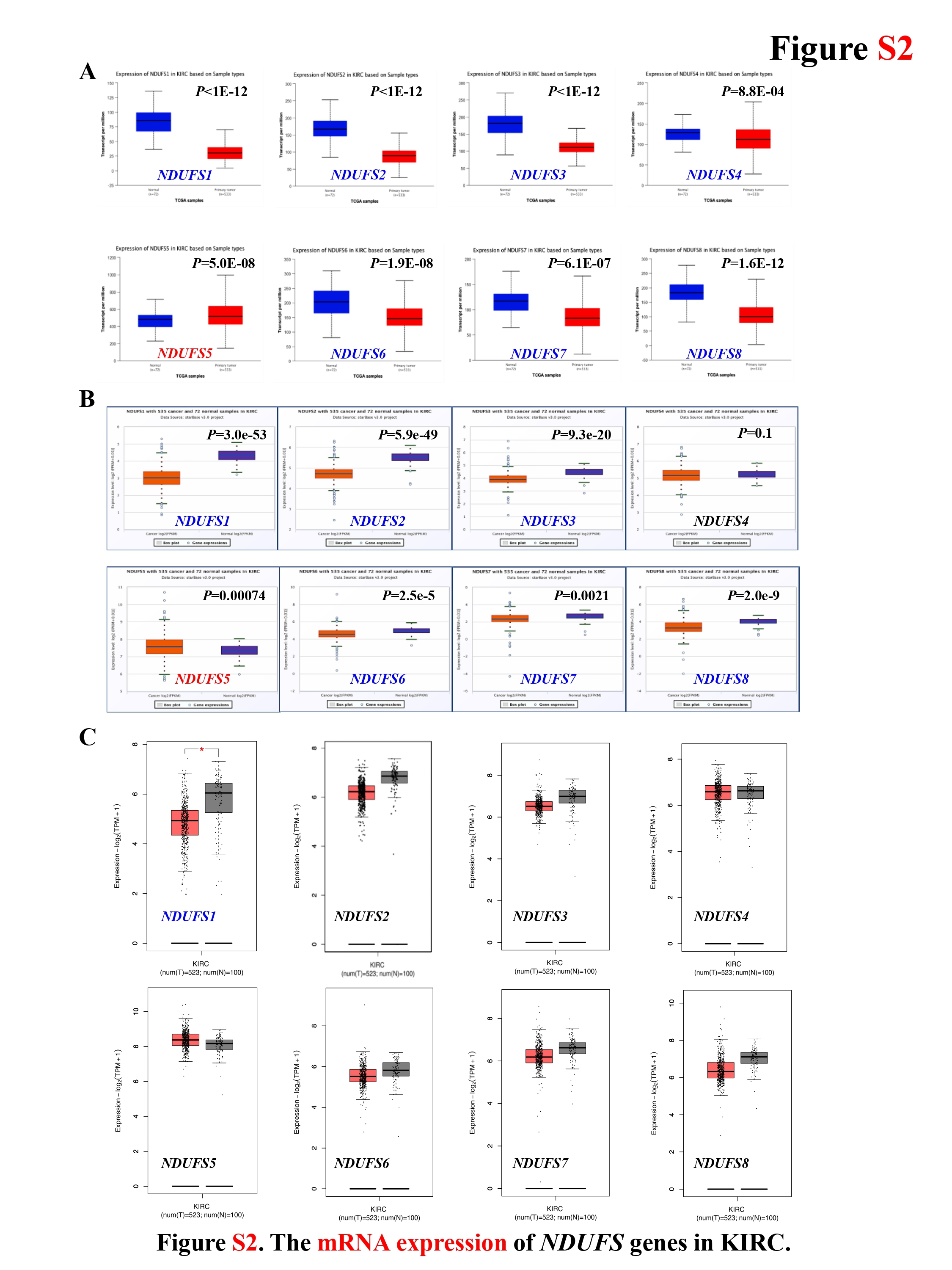

Supplement: Supplementary file 1 [file Presentation1.ZIP › supplementary figures/Figure S2.jpg]

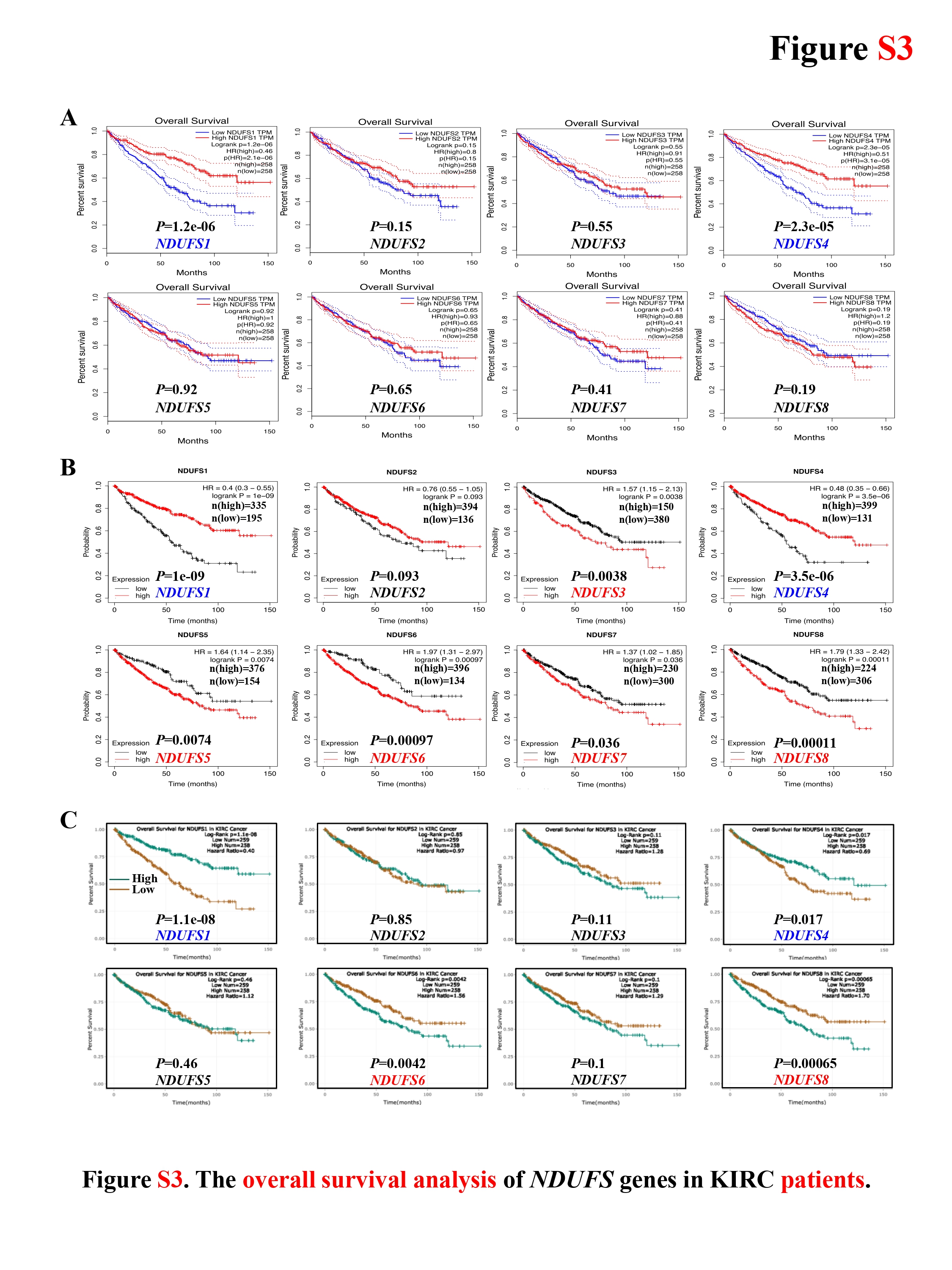

Supplement: Supplementary file 1 [file Presentation1.ZIP › supplementary figures/Figure S3.jpg]

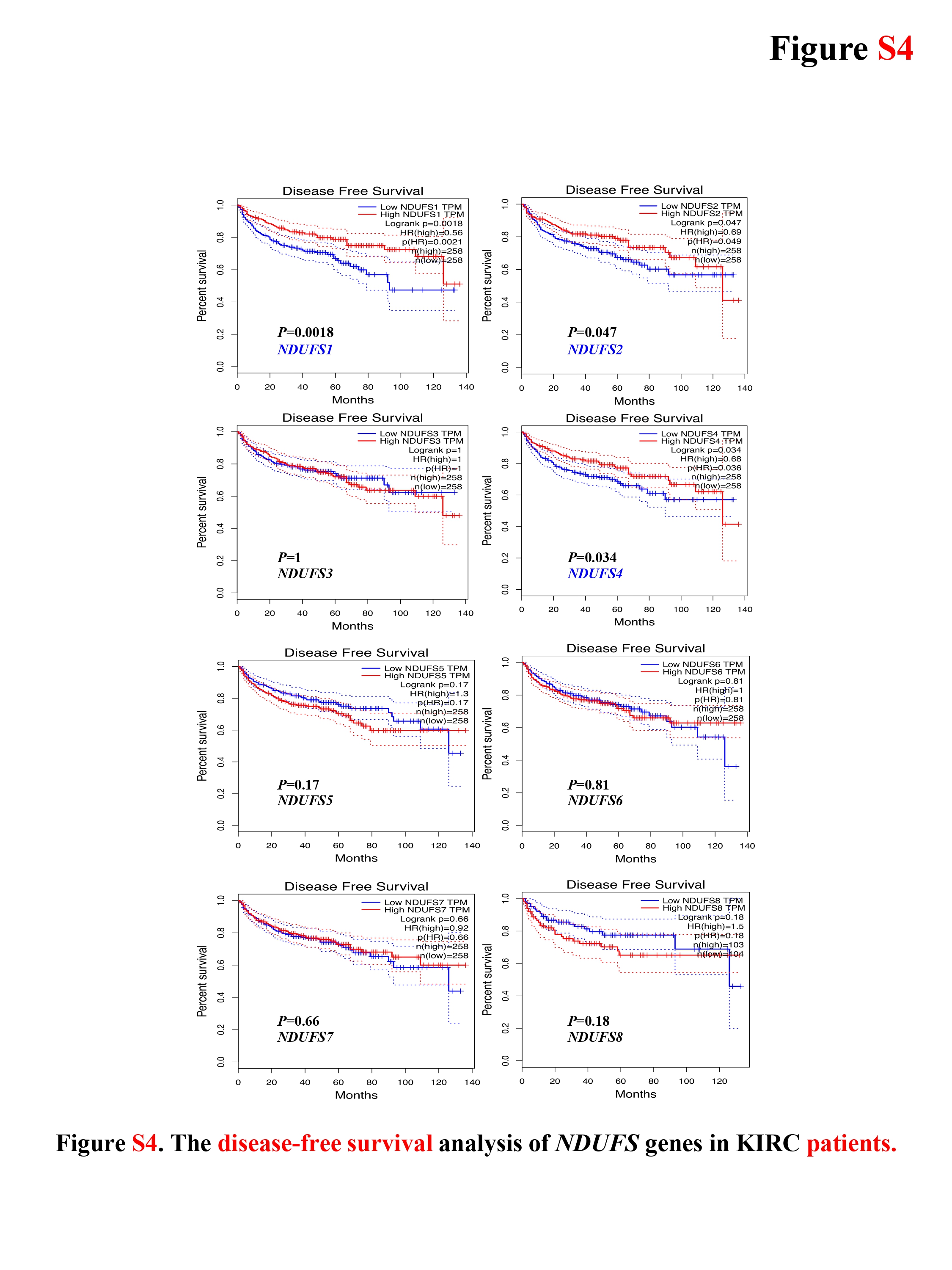

Supplement: Supplementary file 1 [file Presentation1.ZIP › supplementary figures/Figure S4.jpg]

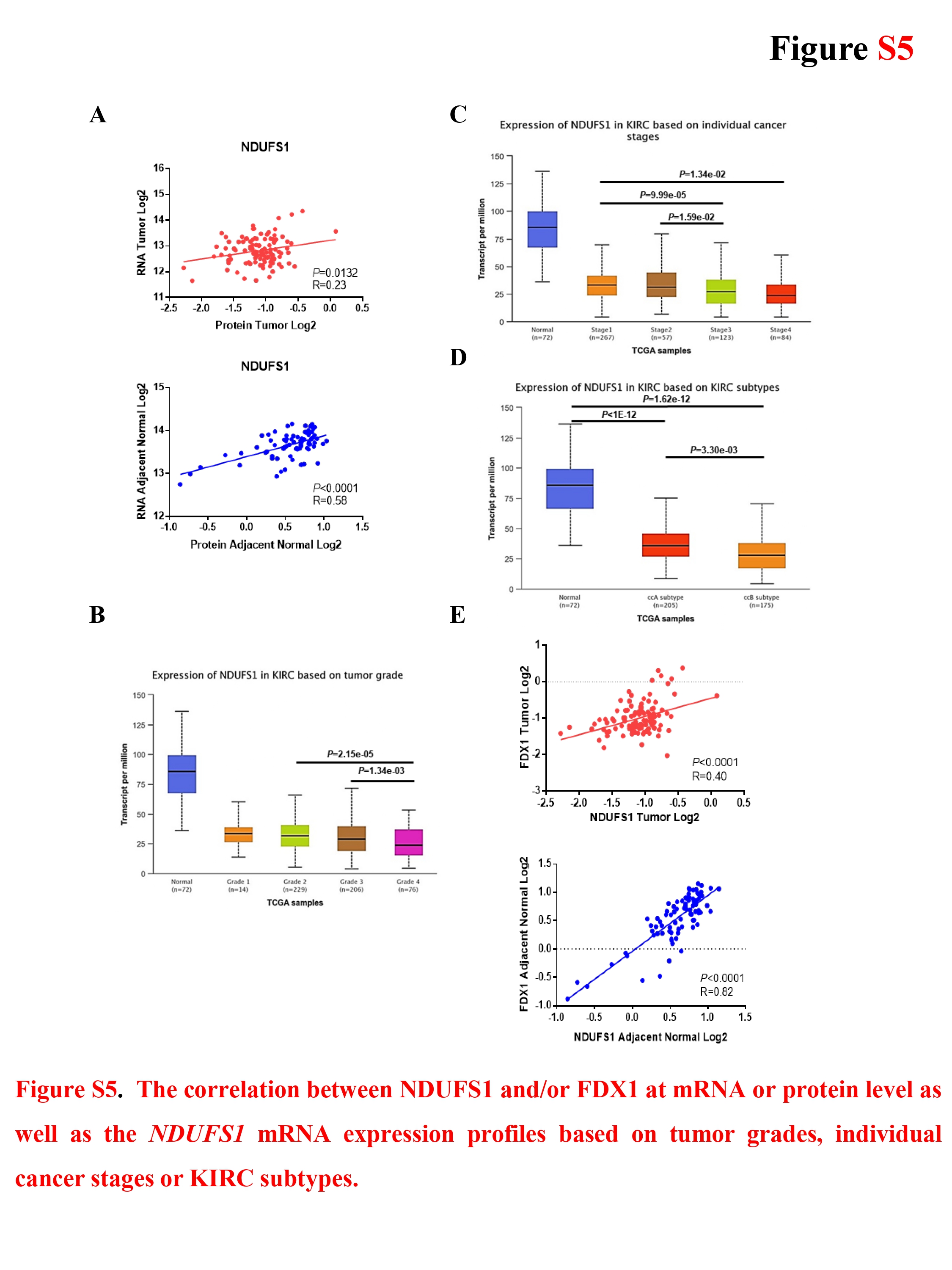

Supplement: Supplementary file 1 [file Presentation1.ZIP › supplementary figures/Figure S5.jpg]

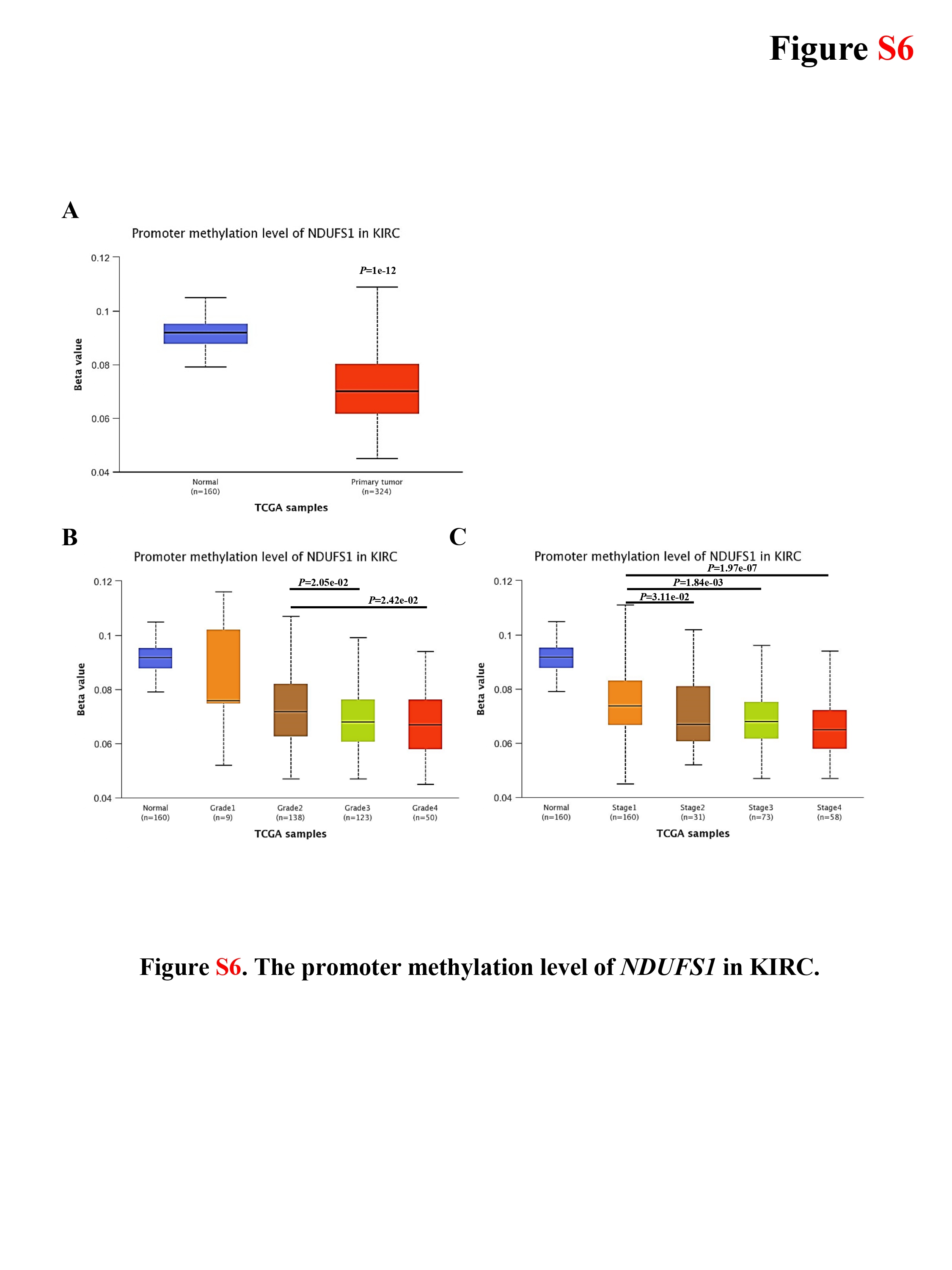

Supplement: Supplementary file 1 [file Presentation1.ZIP › supplementary figures/Figure S6.jpg]
