# Supplementary material for: The combination of NDUFS1 with CD4+ T cell infiltration predicts favorable prognosis in kidney renal clear cell carcinoma
Source: Front Cell Dev Biol. 2023 Jul 4;11:1168462. doi: 10.3389/fcell.2023.1168462 (PMC10352660; doi:10.3389/fcell.2023.1168462)
Supplement: Supplementary file 2 [file DataSheet1.docx]

**Supplemental Figure Legends**

**Figure S1. Flowchart of study design.** K-M Plotter, Kaplan-Meier Plotter**;** OS, overall survival; TMA, tissue microarray; mIF, multiplexed immunofluorescence; TCGA, The Cancer Genome Atlas.

**Figure S2. The mRNA expression of *NDUFS* genes in KIRC.** The mRNA expression of *NDUFS* genes in KIRC tissues as compared to normal tissues were analyzed by the UALCAN **(A)**, ENCORI **(B)** and GEPIA **(C)** database.

**Figure S3. The overall survival analysis of *NDUFS* genes in KIRC patients.** The overall survival analysis in KIRC patients with high or low expression of *NDUFS* genes were analyzed by GEPIA (**A**), Kaplan-Meier Plotter (**B**) and ENCORI (**C**).

**Figure S4. The** **disease-free survival analysis of *NDUFS* genes in KIRC patients.** The disease-free survival analysis in KIRC patients with high or low expression of *NDUFS* genes were analyzed by GEPIA database.

**Figure S5. The correlation between NDUFS1 and/or FDX1 at mRNA or protein level as well as the *NDUFS1* mRNA expression profiles based on tumor grades, individual cancer stages or KIRC subtypes.** (**A**)The correlations between mRNA and protein level of NDUFS1 in KIRC tissues or normal tissues were analyzed by cProSite. (**B-D**) *NDUFS1* mRNA expression based on tumor grades (B), individual cancer stages (C) or KIRC subtypes (D) were analyzed in UALCAN database. (**E**) The correlations between FDX1 and NDUFS1 in KIRC tissues or normal tissues were analyzed by cProSite.

**Figure S6.** **The promoter methylation level of *NDUFS1* in KIRC.** (**A**) The promoter methylation level of *NDUFS1* in KIRC tissues as compared to normal tissues were analyzed by UALCAN database. (**B-C**) The promoter methylation level of *NDUFS1* based on tumor grades (B) or individual cancer stages (C) were analyzed by UALCAN database.
